# *ETS* Related Gene mediated Androgen Receptor Aggregation and Endoplasmic Reticulum Stress in Prostate Cancer Development

**DOI:** 10.1038/s41598-017-01187-4

**Published:** 2017-04-24

**Authors:** Taduru L. Sreenath, Shiela S. Macalindong, Natallia Mikhalkevich, Shashwat Sharad, Ahmed Mohamed, Denise Young, Talaibek Borbiev, Charles Xavier, Rishita Gupta, Muhammad Jamal, Kevin Babcock, Shyh-Han Tan, Marja T. Nevalainen, Albert Dobi, Gyorgy Petrovics, Isabell A. Sesterhenn, Inger L. Rosner, Charles J. Bieberich, Peter Nelson, Valeri Vasioukhin, Shiv Srivastava

**Affiliations:** 1 Center for Prostate Disease Research, USU Walter Reed Department of Surgery, Uniformed Services University of the Health Sciences, Bethesda, MD USA; 2 MCW Cancer Center, Medical College of Wisconsin, Milwaukee, WI USA; 3 Department of Genitourinary Pathology, Joint Pathology Center, Silver Spring, MD USA; 4 Department of Biological Sciences, University of Maryland Baltimore County, Baltimore, MD USA; 5 Division of Human Biology, Fred Hutchinson Cancer Research Center, Seattle, WA USA; 6 Urology Services, Walter Reed National Military Medical Center, Bethesda, MD USA

## Abstract

Mechanistic studies of deregulated ERG in prostate cancer and other cancers continue to enhance its role in cancer biology and its utility as a biomarker and therapeutic target. Here, we show that ERG, through its physical interaction with androgen receptor, induces AR aggregation and endoplasmic reticulum stress in the prostate glands of *ERG* transgenic mice. Histomorphological alterations and the expression of ER stress sensors Atf6, Ire1α, Perk, their downstream effectors Grp78/BiP and eIF2α in *ERG* transgenic mouse prostate glands indicate the presence of chronic ER stress. Transient activation of apoptotic cell death during early age correlated well with the differential regulation of ER stress sensors, in particular Perk. Epithelial cells derived from *ERG* transgenic mouse prostates have increased prostasphere formation with resistance to radiation induced cell death. Continued activation of cell survival factors, Atf6 and Ire1α during chronic ER stress due to presence of ERG in prostate epithelium induces survival pathways and provides a selection pressure in the continuum of ERG dependent neoplastic process. These novel insights will enhance the understanding of the mechanistic functions of ERG in prostate tumor biology and towards development of early targeted therapeutic strategies for prostate cancer.

## Introduction

Prostate cancer continues to be the most commonly diagnosed and a leading cause of cancer deaths in Western countries^1, 2^. Similar to many cancers, prostate cancer mortality has been associated with metastasis. The genetic aberrations associating with the castration-resistant prostate cancer (CRPC), the fatal stage of the disease, are numerous and hereogeneous due to the as a consequence of genomic instability, resulting into abnormal cellular functions^3^. Deregulated androgen receptor (AR) signaling due to either mutations or altered expression of the AR and its cofactors (activators or suppressors) have also been identified as a critical factors in prostate cancer development, progression and metastasis^4^. Mutations of the driver genes, in particular, oncogenes and tumor suppressor genes play a critical role in the initiation of oncogenic process in a cell and subsequently alter the global gene expression patterns^5^.

Oncogenic activation of *ETS R*elated *G*ene (*ERG*) via chromosomal rearrangements has been well established in diverse human cancers such as Ewing sarcoma^6^, acute myeloid leukemia^7, 8^ and over half of all prostate cancers in Western countries^9–12^. ETS related gene (ERG) fusions with androgen regulated gene promoters in prostate cancer patients constitute to a higher percentage in Caucasian men and a much lower frequency in men of African and Asian race^9–11, 13^. *TMPRSS2-ERG* fusion brings *ERG* under androgen-regulated *TMPRSS2* gene promoter which encodes near full length ERG protein products with deletion of 32 amino terminal aminoacids^14^. Since then, several studies have focused on understanding the biological functions of ERG in prostate cancer initiation and progression^14–18^. Transgenic mouse models engineered to express human *ERG* gene in prostate specific manner with modified rat probasin (ARR2PB) promoter showed variable phenotypes including prostate intraepithelial neoplasia (PIN)^14–19^. Despite the less understood mechanistic role of ERG in tumor initiation, these mice developed adenocarcinoma upon the introduction of additional genetic mutations in *PI3K/AKT/PTEN* axis^14, 18, 19^. Moreover, expression of ERG in prostate epithelium resulted in reprogramming of the AR cistrome especially in the presence of *PTEN* inactivation^19^. A recent study showed that, ERG expressing mouse prostates developed adenocarcinoma in older mice through activation of YAP1, a critical component of Hippo pathway^20^.

Since the role of ERG needs to be better understood in early stages of prostate tumorigenesis, we hypothesized that ERG over expression may initiate oncogenic process through activation of cell survival mechanisms, either by abrogating luminal cell differentiation or potential immortalization to provide favorable envornoment for secondary mutations. To test this hypothesis, we focused on mechanistic aspects such as morphological and molecular alterations induced by the overexpression of ERG in prostate epithelium by extensive analysis of both *ERG* transgenic mouse prostate glands and LNCaP cell line transduced with and inducible lentiviral *ERG* construct. In these model systems, one of the most prominent and novel morphological phenotype observed was endoplasmic reticulum (ER) stress. ER stress is a condition that results due to improperly folded secretory and transmembrane proteins due to environmental insults^21^. Further, experiments with lentiviral ERG transduced LNCaP cells showed a physical interaction between ERG and AR, aggregation of AR protein, induction of ER stress response proteins and resistance to cell death. The results presented in this study support our hypothesis and provide a mechanism for how the overexpression of ERG results in AR aggregation, ER stress, apoptosis and eventual cell survival. Importantly, we also establish that the ERG induced ER stress is necessary for developing resistance to cell death towards the initiation of tumorigenic process.

## Results

### Mouse prostate glands expressing Tg*-ERG* display increased cell death due to apoptosis

**Figure 1:**
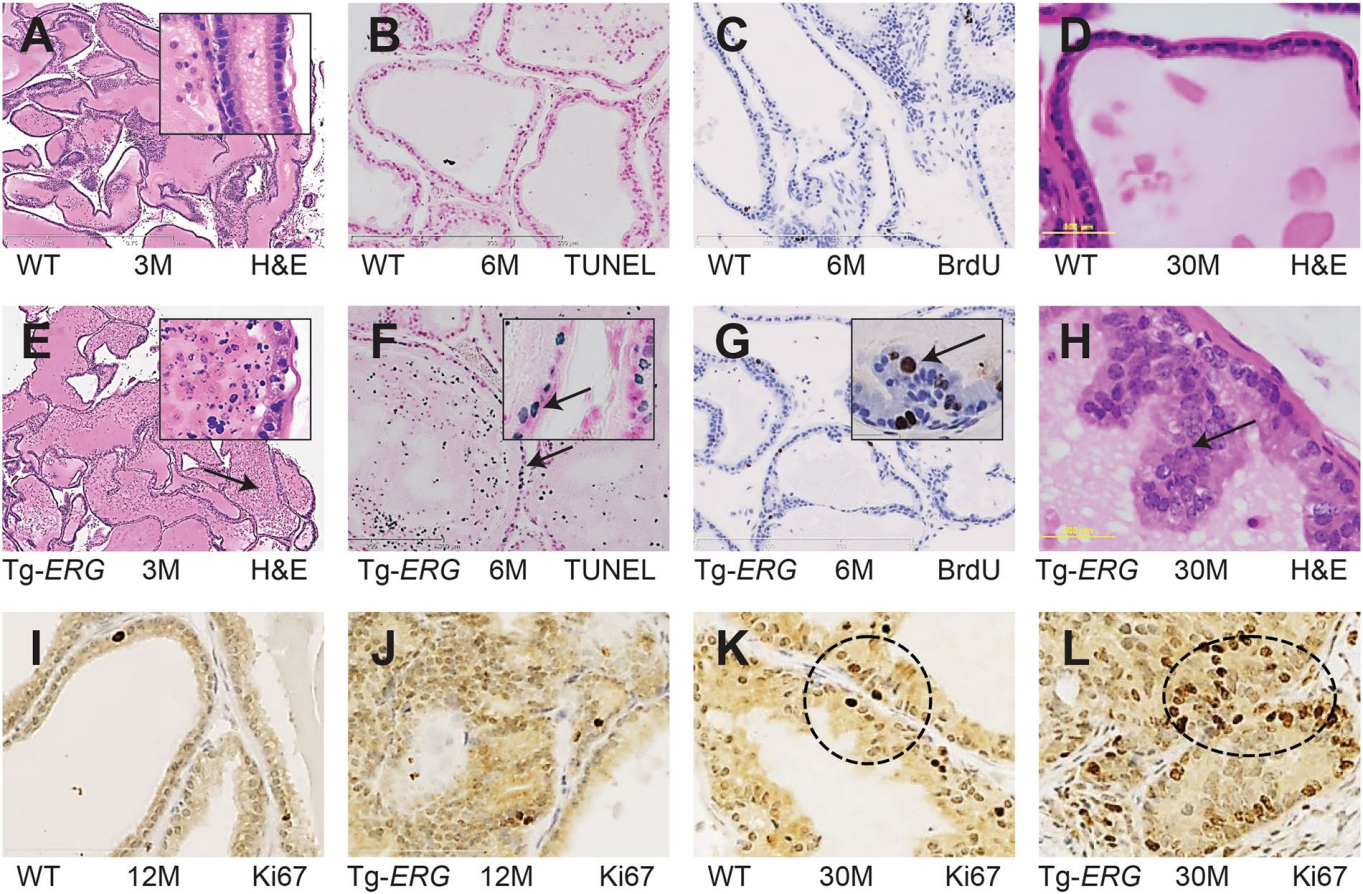
Morphological and histological differences in the ventral prostates of Tg-*ERG* mice. Hematoxylin and eosin staining of prostate glands from 3–30 months-old wild-type (**A,C,G**) and Tg-*ERG* mouse (**B,D,H**) prostate glands. Increased dell death is seen in 3 and 6 months-old display significant cell death (**B,D**). Inserts show nuclear fragmentation and sparsely organized and morphologically distinct luminal epithelial cell layer (note the arrows). TUNEL staining of wild-type (**C**) and Tg-*ERG* (**D**) prostates confirm the cell death due to apoptosis. Short arrow (insert) points to an intact luminal cell undergoing apoptotic cell death while long arrow points to fragmented nuclei of dead cells in the lumen. Cell proliferation analysis has shown an increase in the number of BrdU-positive cells in Tg *ERG* mouse (**F**) than wild-type (**E**). Hematoxylin and eosin staining of 30 month-old transgenic mice display clustered luminal cells resembling high grade PIN lesions (**H**). Similarly, Ki67 immunostaining was also increased in Tg-*ERG* mouse (**J,L**) than wild-type (**I,K**). There is an increase in the Ki67 staining pattern in 30 month-old mouse prostates than 12 month-old prostates (**I–L**). Total number of mice used for each analysis is 5 (n = 5).

Prostate luminal epithelial cell targeted ERG transgenic mice, Tg (*Pbsn-ERG) 1Vv* [Tg-*ERG*]^17, 20^ were generated by a widely used rat probasin promoter ARR2PB^22^. Our comprehensive evaluations of the ERG protein in Tg-*ERG* mouse prostate glands revealed higher expression in ventral prostate glands compared to other lobes (ventral > lateral > dorsal > anterior) **(**Suppl. S1). The ERG protein levels appear to be relatively higher in the distal half of the prostate compared to proximal half of the prostate glands **(**Suppl. S1). Consistent with earlier reports^16, 17^, we also noted a significant increase of PIN lesions with increasing age of the Tg-*ERG* mice. To address, if variable phenotype in earlier reported *ERG* transgenic mouse prostates was due to low ERG protein expression, we compared levels of ERG protein in adult *ERG* transgenic mouse^17^ prostate glands with human prostate cancer specimens. We did not find any significant differences in the expression levels of ERG protein by immunohistochemistry (IHC) between Tg-*ERG* mouse prostate glands and ERG positive human prostate tumors (Suppl. S2A,B). A novel and prominent histological phenotype observed in the Tg-*ERG* mouse prostates was the increased nuclear fragmentation of luminal epithelial cells (Fig. 1B) compared to their wild-type litter-mates (Fig. 1A). The nuclear fragmentation was confirmed by TUNEL staining suggesting that the luminal epithelial cell death was due to apoptosis, which was persistent until 12 months, with the highest levels around 6 months of age (Fig. 1C D; Suppl. S3A–G). It is well established that oncogenic signals induce cellular stress responses, including metabolic stress, apoptosis, DNA damage responses, and cellular senescence^23, 24^. Apoptotic cell death in Tg-*ERG* mouse prostate glands was most frequently observed in the ventral, less so in dorso-lateral, and rarely in the anterior lobes. Therefore, we focused subsequent studies on ventral prostate associated cell death and survival events. The increased incidence of apoptosis in prostate ducts of Tg-*ERG* mice suggested for a well-established androgen signaling mediated differentiation state of rodent prostates^25^. To examine if the surviving epithelial cells facing the lumen have proliferative properties, the Tg-*ERG* mice analyzed for BrdU incorporation in DNA (Fig. 1E,F; Suppl. S4A–G). Histological examination revealed that the luminal epithelial cells from 30 month-old prostates exhibited altered nuclear morphology and pockets of increased clusters high grade PIN lesions (Fig. 1G,H). A small but, significant number of proliferating cells were observed in the luminal compartment of 12 month-old ERG transgenic mouse prostates which further increased in 30 month-old Tg-*ERG* mice **(**Fig. 1I–L; Suppl. S5A,B **)**.

### Luminal epithelial cells with Tg*-ERG* expression display abnormal ER and ER stress

**Figure 2:**
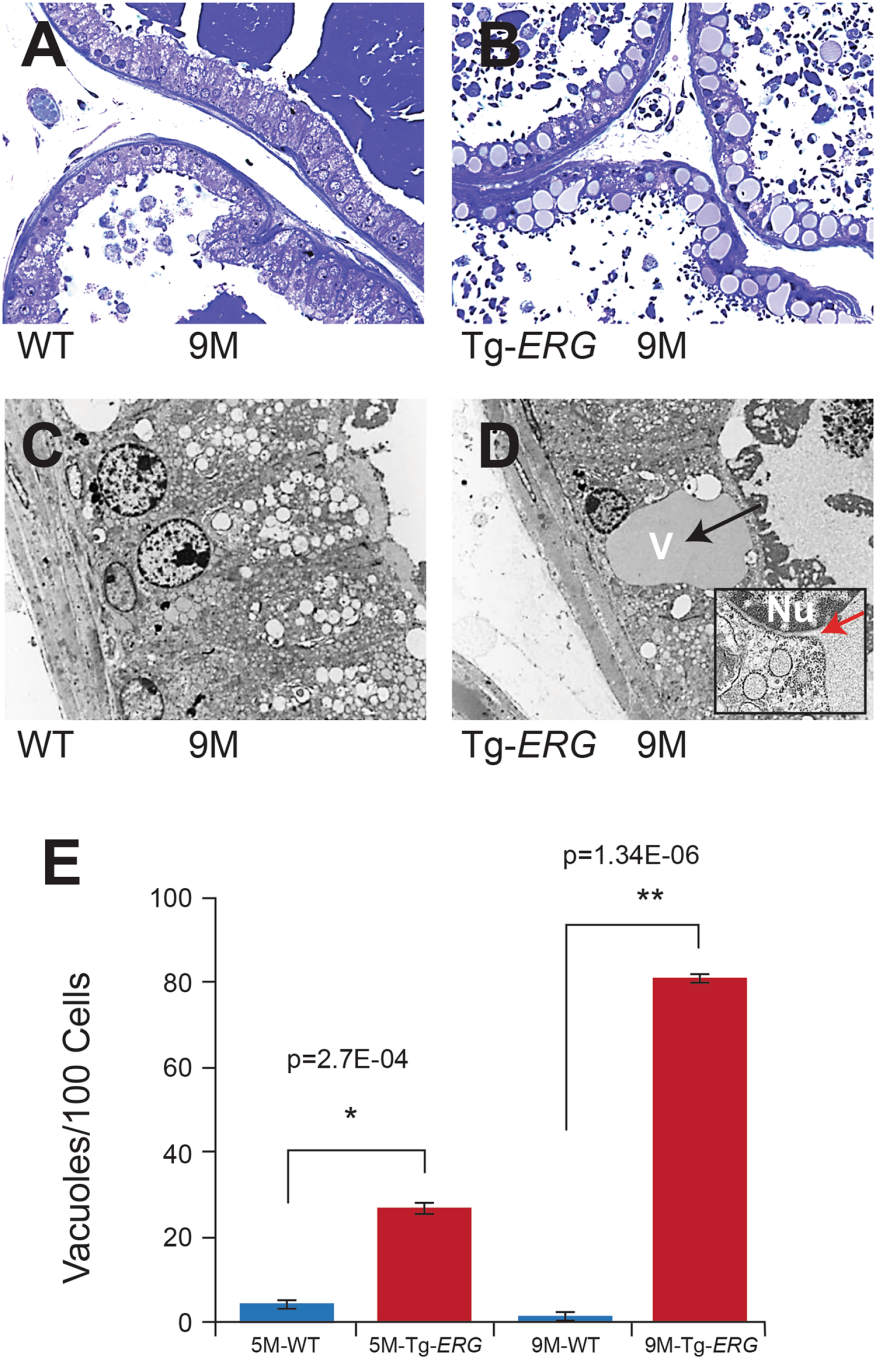
ERG induces histo-morphological phenotype in mouse prostate glands. Toluidine blue stained plastic sections of wild-type mouse prostate glands (**A**) display well-organized, terminally differentiated, tall columnar luminal epithelial cells with basally positioned nuclei. Tg-*ERG* mouse ventral prostate (**B**) luminal epithelial cells displayed cuboidal cells with irregularly bordered nuclei and increased vacuolization. TEM analysis of the wild-type (**C**) ventral prostates displayed tall columnar luminal cells, basal nuclei and large number of clear secretory vesicles in the apical cytoplasm. Tg-*ERG* mouse prostate epithelial cells (**D**) displayed an abnormally large vacuole adjacent to nuclei (red arrow showing the extension of nuclear envelop into ER). The quantitative analysis of morphological changes (appearance of vacuoles in the Tg-*ERG* mouse prostates) in the luminal epithelial cells was performed on Toluidine blue stained plastic sections of Tg-*ERG* and wild-type prostate glands of 5 and 9 months-old mice (**E**).

Tg-*ERG* prostates from 3 to 12 month-old mice processed in methyl-methacrylate for plastic sections stained with toludine blue showed increased vacuolization and altered morphology (arrows Fig. 2B) compared to the wild-type controls (Fig. 2A). Subsequent ultra-structural analysis of Tg-*ERG* mouse prostate glands by TEM displayed significant cytoplasmic abnormalities such as ballooned vacuoles of various sizes with filamentous content (Fig. 2C,D) that were similar to the morphological features of ER stress^26^. The most striking morphological difference observed was abnormally swollen ER (arrow Fig. 2D). In wild-type mouse prostate glands, the cellular cytoplasm contained flattened rough endoplasmic reticulum (ER) cisternae and secretory vesicles with different electro-densities in the apical region. The presence of the abnormally swollen ER (vacuoles) were found increased significantly in 9 months-old mouse prostates (Fig. 2E). These morphological observations of balooned ER appeared to be ERG specific and were not detected in Lo*MYC* transgenic mouse prostates (Suppl. S6). Taken together these data suggested a dysfunctional ER in the presence of *ERG* overexpression.

**Figure 3:**
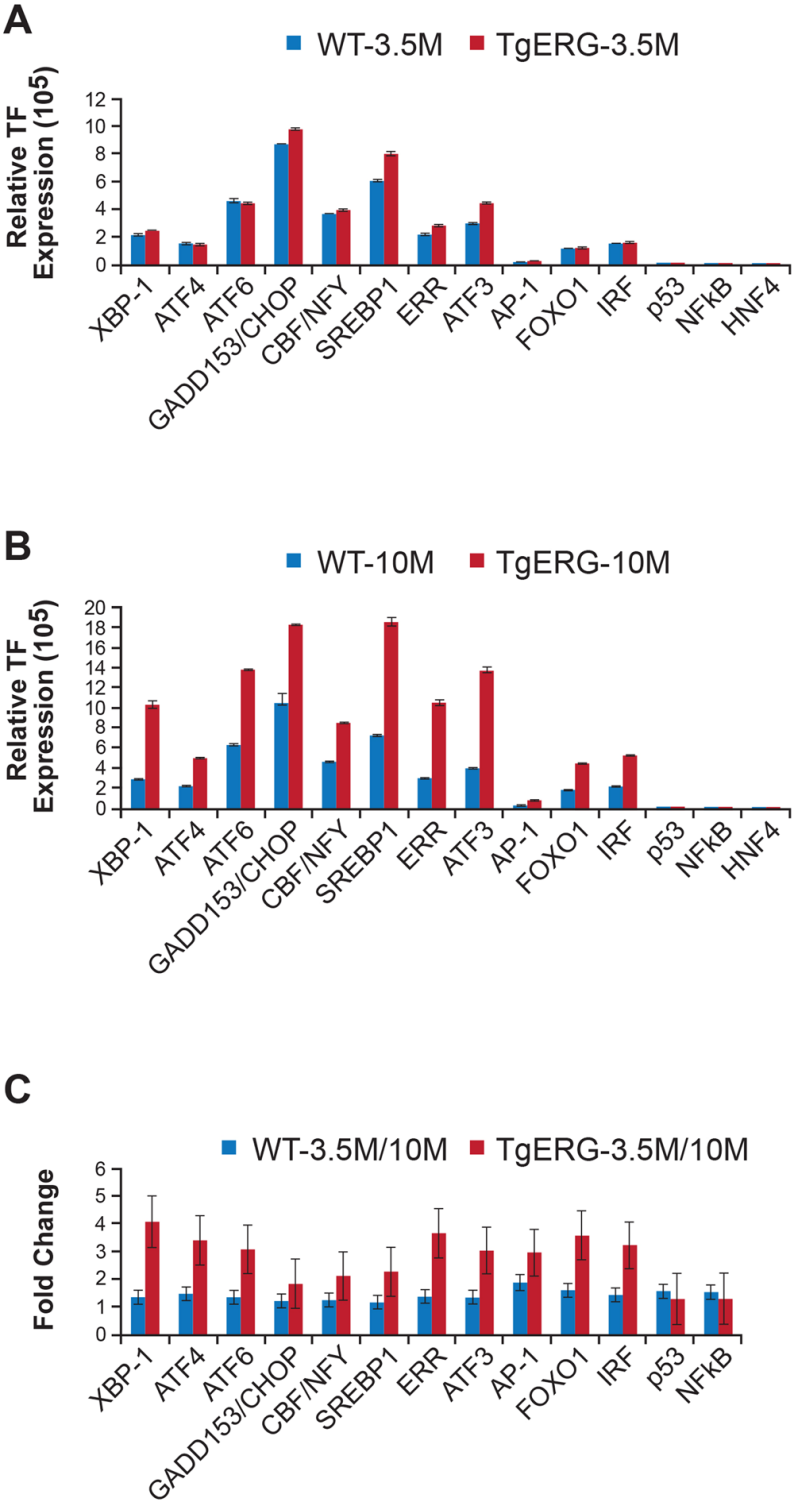
ERG deregulates ER stress transcription factors. ER Stress (UPR) TF Activation Profiling Plate Array was used to analyze transcription factors (TFs) in the prostates of 3.5 and 10 month-old Tg*-ERG* mice compared to wild-type mice (**A,B**). Activity of ER transcription factors Xbp1, Gadd153/Chop, Cbf/Nfy, Serbp1, Err, Atf3 were slightly up regulated in 3.5 month-old prostates (**A**). However, by 10 months of age, Xbp1, Atf4, Atf6, Gadd153/Chop, Cbf/Nfy, Srebp1, Err, Atf3, Foxo1, Irf were up regulated significantly (**B**). Expression of ER stress transcription factors in 10 month-old prostates from Tg-*ERG* showed significant increase compared to 3 months old prostates from Tg-*ERG* (**C**).

**Figure 4:**
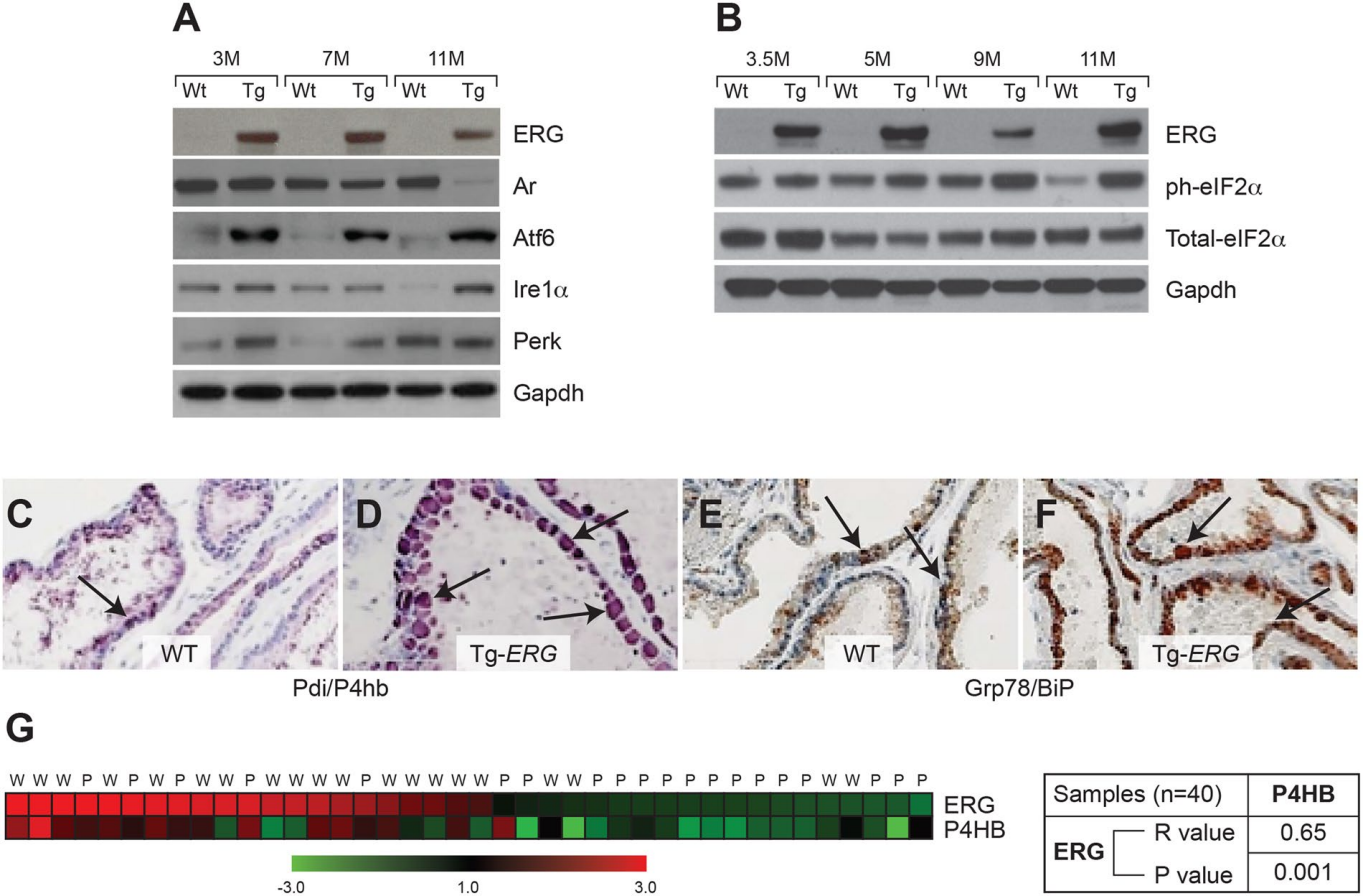
Regulation of ER stress sensors by ERG in mouse prostates. Western blot analysis  **(A)** of ERG, Ar and ER stress sensors Atf6, Ire1α and Perk show increased expression in 3, 7 and 11 month-old Tg-ERG mouse prostates. Ar expression appears to decrease in the Tg-*ERG* prostates and is more significant in older mice. Similarly, there is an increase in the phosphorylation of eIF2α, a down stream target for Perk, in the transgenic mouse prostate glands (**B**). Immunohistochemical analysis of and P4hb/Pdi (**C,D**) and Grp78/BiP (**E,F**) in wild-type (**C,E**) and Tg-*ERG* (**D,F**) mouse prostates show elevated expression. Analysis of ERG and P4HB expression (**G**) in human prostate has shown significant correlation (***n***) (r = 0.65; P = 0.001). Expression data obtained from an 80GeneChip microarray show up-regulation as red and down-regulation as green.

**Figure 5:**
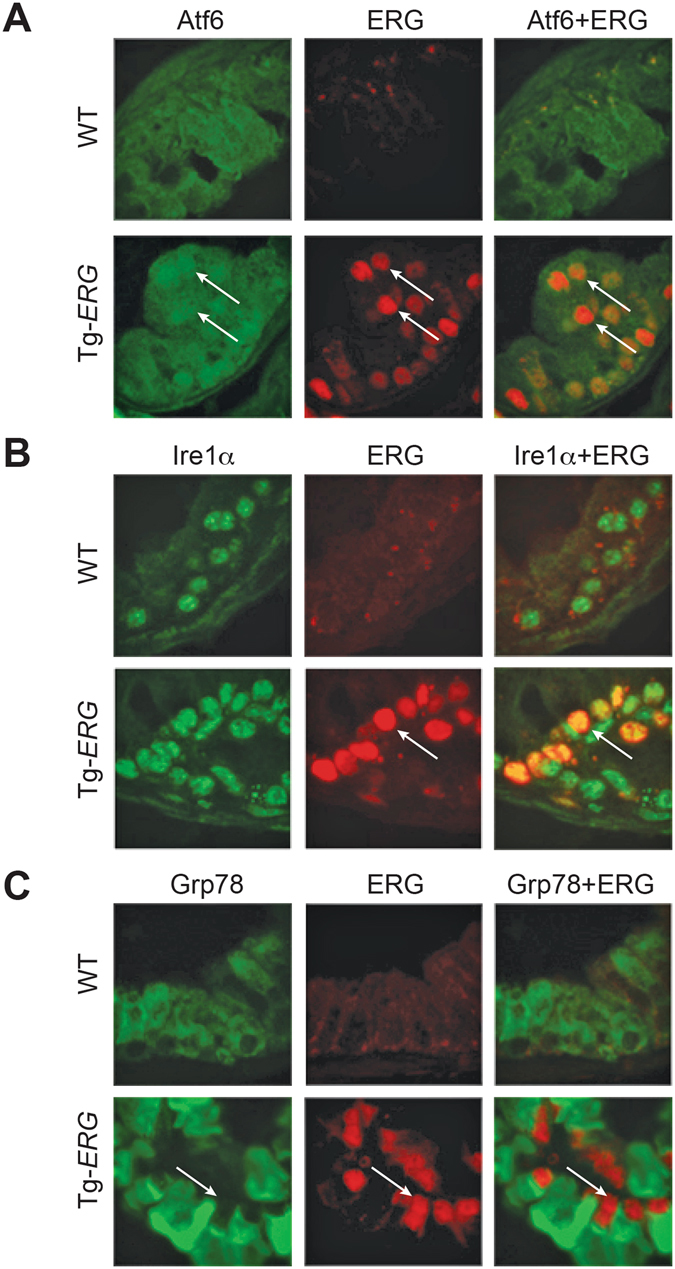
Colocalization of ERG with ER stress sensors Atf6, Ire1α and ER chaperone protein Grp78/BiP by indirect immunofluorescence in 9 month-old Tg-*ERG* mouse prostate glands. Immunolocalization of Atf6 reveals the presence of activated Atf6 in the nucleus of Tg-*ERG* prostate luminal epithelial cells along with colocalized ERG (white arrows in the lower panel of **A**). Ire1α expression was increased in the Tg-*ERG* mouse prostates, correlating well with the colocalized expression of ERG in the luminal epithelial cells (white arrows in the lower panel of **B**). ER resident chaperone protein Grp78/BiP,  localized mainly in the cytoplasm of the luminal epithelial cells was increased in Tg-*ERG* mouse prostates (white arrows in the lower panel of **C**).

In order to gain insights into ER stress and its effect on the stress related transcription factors, initially we performed a qualitative analysis of transcription factor activation profiling plate array assay using protein extracts from Tg-*ERG* mice and wild-type controls. Prostates of 3.5 month-old Tg-*ERG* mice showed slight up regulation of Xbp1, Gadd153/Chop, Cbf/Nfy, Serbp1, Err, Atf3 (Fig. 3A) and the magnitude of the activation of these TFs was significantly higher by 10 months of age (Fig. 3B,C). In addition, other transcription factors such as Atf4, Atf6, Atf3, and Foxo1 were also found elevated in 10 month-old Tg-*ERG* mouse prostates (Fig. 3B). Further, the protein extracts from prostates from 3, 7 and 11 months-old Tg-*ERG* mice exhibited increased expression of critical ER stress sensors proteins: Atf6, Ire1α and Perk (Fig. 4A). Activated Perk mediated phosphorylation of eIF2α showed upregulation in Tg-*ERG* mouse prostates (Fig. 4B). As a consequence of activated ER sensors, UPR proteins such as Pdi/P4hb and Grp78, were upregulated in Tg-*ERG* mouse prostates (Fig. 4D,F). In order to extend the observations of ERG associated UPR induction from *ERG* transgenic mice to human prostate tumors with *ERG* activation, we assessed the correlation of *ERG* and *PDI/P4HB* expression in our Affymetrix GeneChip dataset (NCBI, GEO: GSE32448)^11^. Similar to the expression of P4hb/Pdi in Tg-*ERG* mouse prostates, a significant correlation of *P4HB/PDI* up-regulation was observed in *ERG* positive human prostate tumors (Fig. 4G). We further examined the colocalization of ERG with the ER stress sensors in the Tg-*ERG* mouse prostates. Immunofluorescence analysis revealed the nuclear localization of Atf6 suggesting the activation of Atf6 in the prostate epithelium in Tg-*ERG* mice (Fig. 5A). Increased xpression of Ire1α and Grp78 were also observed in the prostate epithelial cells of Tg-ERG mice (Fig. 5B,C).

### ERG induces ER stress through AR mis-folding/aggregation

**Figure 6:**
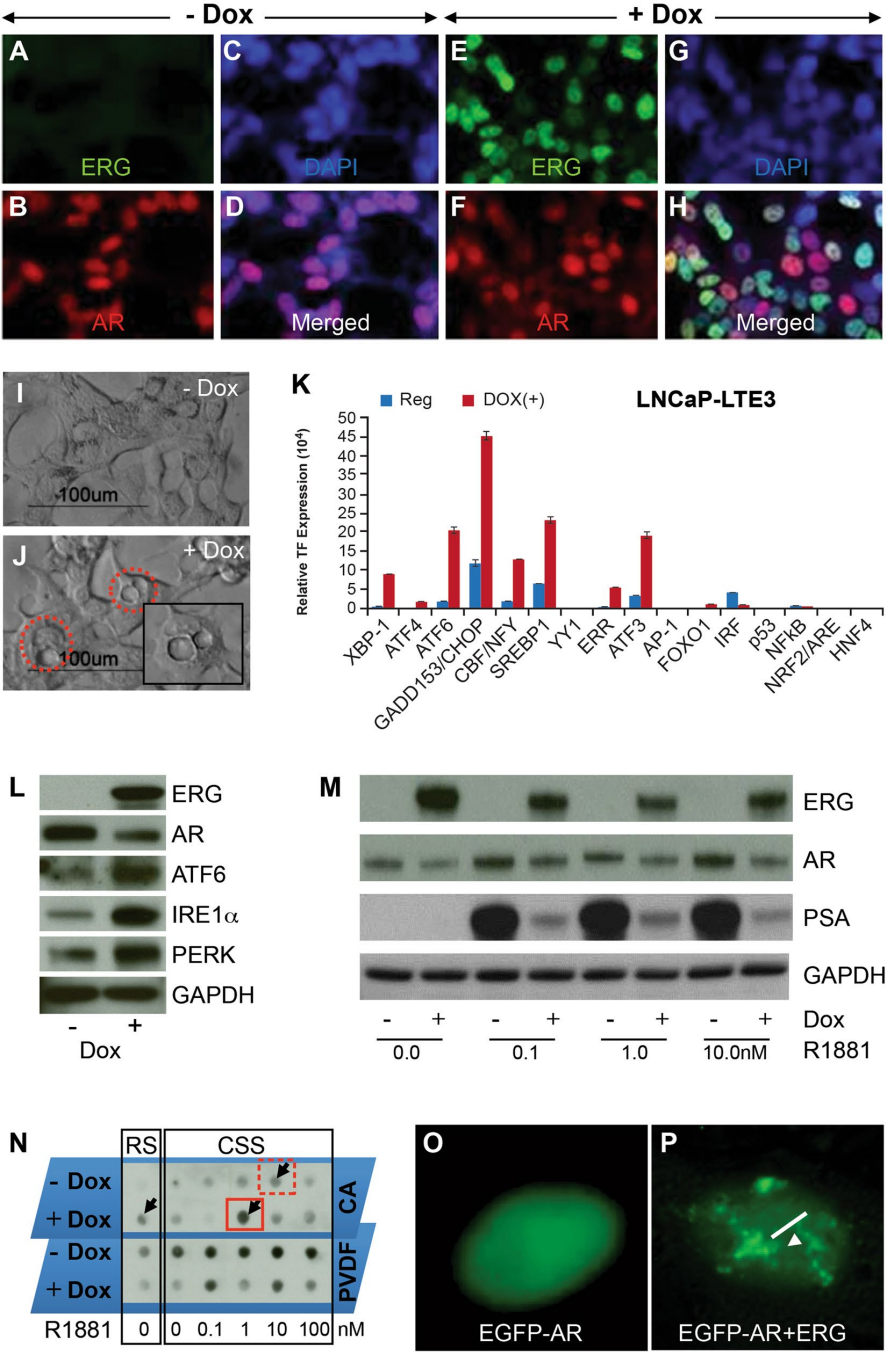
ERG induces ER stress and AR aggregation in LNCaP cells. Stable and doxycycline inducible ERG expressing LNCaP-Ln*TE3* cells were generated by lentiviral expression vector and characterized by immunofluorescence using mouse monoclonal ERG antibodies (9FY) and anti-AR antibodies (**A–H**). ERG expression was detected upon induction with 1 ug/ml of doxycycline treatment for 24 hrs. Note the ERG expression in un-induced (**A**) and induced (**B**). Increased numbers of vacuole formations (red dotted circles) were observed with doxycycline induction of ERG (**J** and Supplementary Figure S7). Analysis of the ER stress transcription factors was analyzed in LNCaP-Ln*TE3* cell lysates prepared from without and with induction of ERG by doxycycline. Levels of XBP1, ATF4, ATF6, GADD153/CHOP, CBF/NFY, SREBP1, ERR, ATF3 were increased with induced ERG (**K**). Western blot analysis showing enhancement of critical ER sensors such as ATF6, IRE1α and PERK (**L**). Induced expression of ERG in the presence of synthetic androgen, R1881 showing altered regulation of AR target genes PSA (**M**). AR aggregation was observed in cells treated with 10% FBS in the presence of induced ERG (**N**; shown by black small arrow). PVDF membrane, which traps monomers shows inverse AR signal with doxycycline induced ERG at 1.0 nm concentration of R1881 (**N**; red solid box). However, higher AR aggregation at 10.0 nM of R1881in the absence of ERG induction was (red dotted box) observed. AR aggregation is shown in LNCaP-Ln*TE3* cells transfected with pEGFP-C1-AR (**O,P**). p*EGFP-C1-AR* transfected LNCaP-Ln*TE3* cells were grown without (**O**) and with (**P**) doxycycline induction showing aggregates.

**Figure 7:**
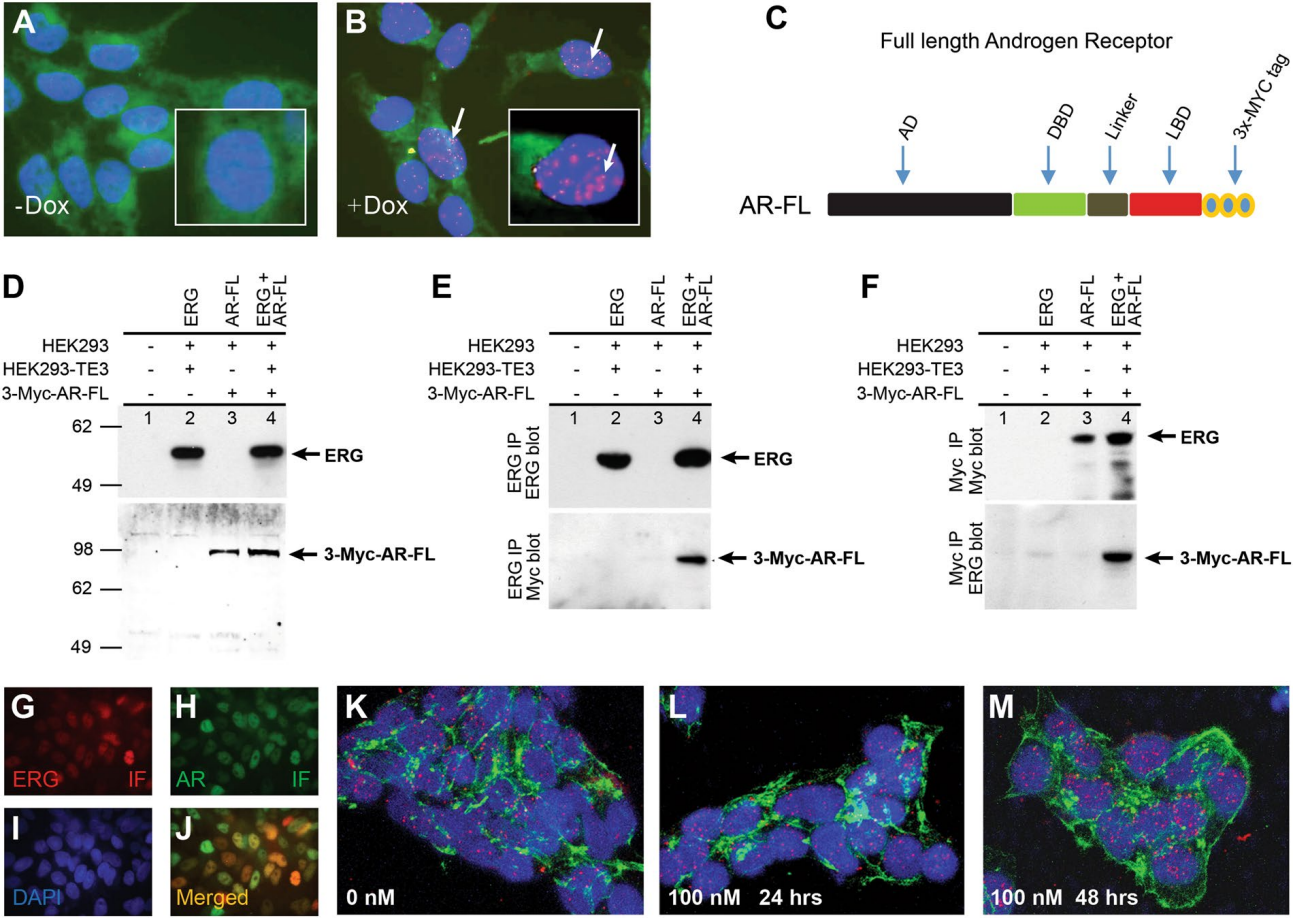
ERG physically interacts with Androgen Receptor. Proximity ligation assay (PLA) on LNCaP-LnTE3 cells without (**A**) or with (**B**) induction of ERG shows a positive interaction with AR protein. Graphical representation of 3x-MYC tagged AR full length and AR deletion construct (**C**) used to transfect HEK293 and HEK293-TE3 cells. HEK-293 and ERG expressing HEK 293-LTE3 cells grown in serum-starved conditions for 20 hours were transfected with full length AR and ERG constructs and stimulated with 1 nM R1881 for another 24 hrs. Immunoblot of cell lysates used in co-IP experiment were tested with anti-ERG MAb and anti-MYC-tag antibody as controls (**D**). ERG interactions with AR were detected by immunoprecipitation of ERG followed by westernblot with anti-ERG MAb (9FY) with mouse polyclonal IgG as control and immunoblotted with rabbit polyclonal anti-MYC-tag antibody (**E**). As a second approach, AR was immunoprecipitated with anti-MYC-tag antibody with rabbit polyclonal IgG as controll and immunoblotted with anti-ERG MAb (9FY) antibody (**F**). To further analyze if the interactions of ERG with AR are dependent on AR dimers or monomers, VCaP cells were grown on cover glasses in the presence of 100 mM resverotrol for 48 hours and PLA was performed to detect changes in physical interactions. Immunoflurescence analysis of ERG and AR in VCaP cells grown for 48 hrs showed colocalization (**G–J**). ERG-AR interactions by PLA showed the presence of interactions at 48 hrs resveratrol treated VCaP cells (**K–M**).

In order to further validate ERG induced ER stress, we generated and characterized a stable cell line (lentivirus transfectants of LNCaP, LNCaP-LnTE3) with doxycycline inducible ERG3 (Fig. 6A–J). LNCaP-LnTE3 cells grown in charcoal-stripped serum (CSS) with subsequent addition of synthetic androgen, R1881(1 nM) and doxycycline ﻿(1 μg/ml) induced ERG expression displayed significant increase in the number of vacuoles (Fig. 6I,J). LNCaP-Ln*TE3* cells grown in regular media (supplemented with 10% FBS) upon doxycline induced ERG expression also displayed stress related phenotype of vacuole formation. ER stress transcription factor activation profiling plate array assay using protein extracts from ERG induced LNCaP-Ln*TE3* cells showed increased activity of transcription factors: XBP1, ATF6, GADD153/CHOP, SREBP1, ERR and ATF3 (Fig. 6K). Further, increased expression of ER sensor proteins, ATF6, IRE1α and PERK were noted in response to ERG induction (Fig. 6L). Consistent with reduced levels of Ar protein detected in Tg-*ERG* prostates (Fig. 4A), LNCaP-LnTE3 cells displayed reduced levels of AR protein in response to ERG induction (Fig. 6M). To further evaluate AR or AR regulated gene expression in the presence of ERG, we analyzed a well characterized AR target gene, PSA in LNCaP-Ln*TE3* cells (Fig. 6M). This observation was consistent with the previous findings of ERG mediated attenuation of AR-regulated PSA expression in *TMPRSS2-ERG* positive VCaP cells^27, 28^. These results indicated that ERG may interfere with the functions of AR, such as maintenance of differentiation or survival of terminally differentiated luminal epithelial cells. It has been postulated that the AR inclusions or aggregation can be induced due to physiological fluctuations such as differentiation or stress disturbances by androgens resulting into activation of ER stress^29^. Mechanistic studies have shown a link between AR aggregation and UPR leading to the development of Kennedy disease^30^. Higher concentrations of androgens are also known to aggregate AR protein leading to ER stress and cell death^29, 31^. Since AR is a critical molecule in development of prostate glands and disease conditions, we examined the AR aggregation by filter aggregation assay^32^ in the presence or absence of ERG to assess if the AR mis-folding/aggregation plays role in ERG induced ER stress. Lower R1881 concentration (1.0 nM) in the presence of ERG induction resulted in AR aggregation, comparable to a higher R1881 concentration alone (10 nM), suggesting for comparable cellular stress induced by ERG or higher R1881 concentrations (Fig. 6N). ERG in the presence of 1.0 nm R1881 appears to induce AR aggregates in a synergistic manner. Further, LNCaP-LnTE3 cells transfected with pEGFP-C1-AR^33^ showed nuclear aggregation of AR protein upon ERG induction. (arrow Fig. 6P) compared to controls (Fig. 6O). To assess the potential of physical interaction between ERG and AR that may be critical in impairing differentiation or compromising the AR mediated functions, we utilized proximity ligation assay (PLA) in LNCaP-Ln*TE3* cells to see the interactions *in situ*. We observed a significant interaction between ERG and AR within the native chromatin context (Fig. 7B) only with the induction of ERG by doxycycline treatment. We further examined AR interaction with ERG by utilizing the construct of 3xMYC tagged AR^34^. HEK293 and a stable cell line of HEK293 with constitutive expression of ERG (HEK293-TE3) were transfected with 3xMyc-AR-FL construct (Fig. 7C). Both ERG and AR-FL were expressed and were detected with anti-ERG antibodies (9FY) and Myc-Tag antibodies respectively (Fig. 7D). Immunoprecipitation of ERG followed by immunoblotting of Myc-AR revealed a significant interaction with ERG (Fig. 7E). Similar results were obtained with immunoprecipitation with Myc-tag antibodies followed by immunoblotting with ERG antibodies (Fig. 7F). Since AR was shown to for homodimers, we further examined if the ERG interactions with AR are specific to either the AR-homodimer or AR-monomer by treating TMPRSS2-ERG harboring VCaP cells with AR dimerization inhibotor (resveratrol)^35^. Immunoflurescence assay shows the colocalized expression of ERG and AR in the nuclei of VCaP cells (Fig. 7G–J). Results from PLA show significant ERG-AR interactions in the presence of 100 mM resveratrol suggesting that ERG interacts with AR monomers upto 48 hrs (Fig. 7K–M).

### Luminal epithelial cells isolated from ERG transgenic mouse prostates show altered expression of cell surface markers, sphere forming capability and differentiation

**Figure 8:**
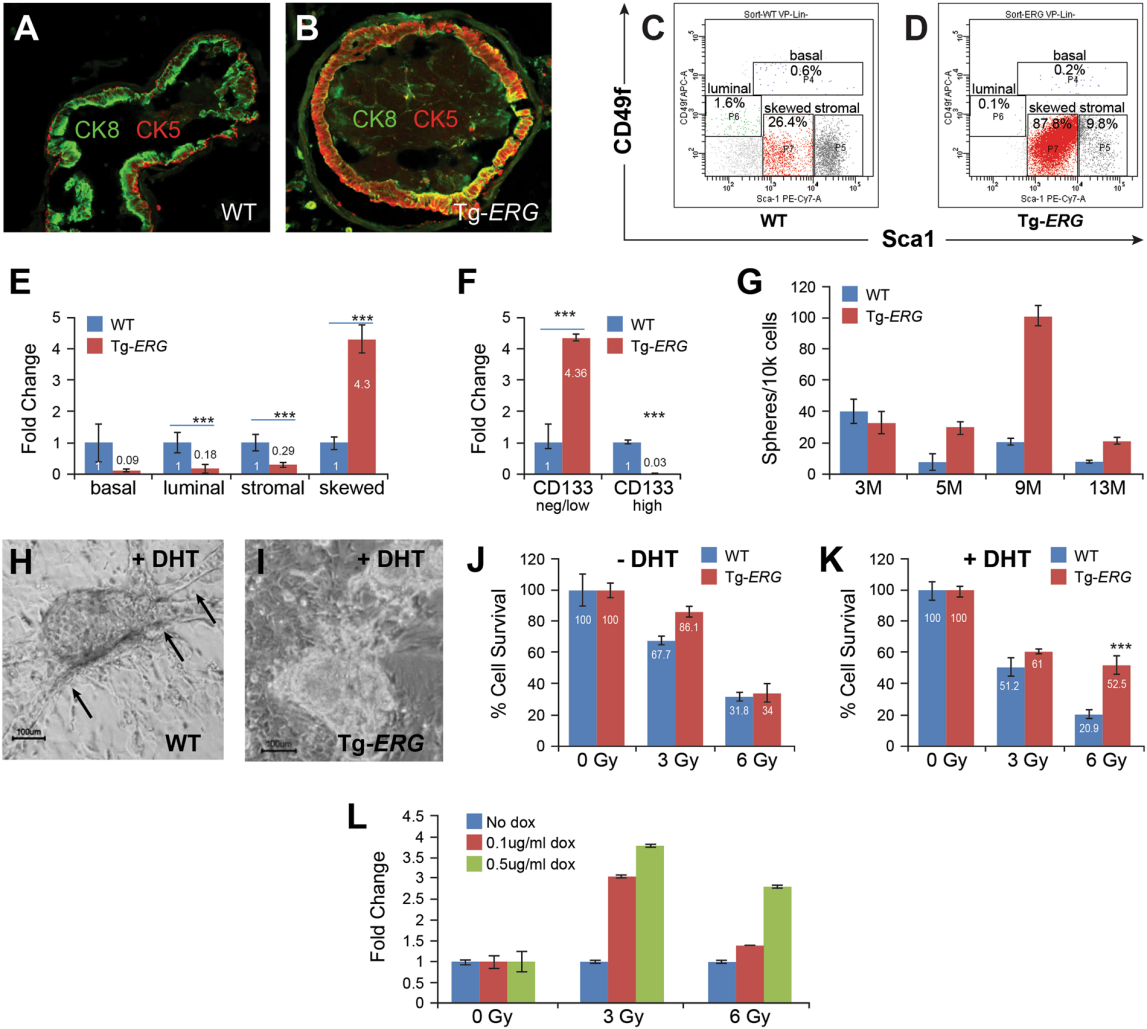
ERG alters molecular and morphological characteristics of luminal epithelial cells. Expression of Ck5 (basal) and Ck8 (luminal) cell markers in wild-type and Tg-*ERG* mouse prostates was performed by immuno-fluorescence on frozen sections. Wild-type mouse prostate glands express Ck5 (red) and Ck8 (green) (**A**). Tg-*ERG* prostate glands express increased number of cells double positive for both Ck5 and Ck8 (**B**). FACS analysis of isolated luminal cells from wild-type (**C**) and Tg-*ERG* mice (**D**) showed a significant increase in CD49f (low) and Sca-1 (med) in Tg-*ERG* mice. Normalized fold induction/reduction in frequencies of basal, luminal, stromal, skewed populations show about 4-fold increase of skewed population in Tg-*ERG* prostates (**E**). The frequencies of CD133 expression in percentages within skewed (red), stromal (grey) populations in wild-type and Tg-*ERG* mice show increase in CD133^negative/low^ and decrease in CD133^high^ cells in Tg-*ERG* prostates (**F**). Quantitative analysis of sphere forming units (**G**) from ventral prostates of wild-type and Tg-*ERG* mice shows the number of spheres grown per 10^4^ input cells. The number of sphere forming units (SFU)/10,000 cells in the Tg-*ERG* was approximately 1–5 fold compared to controls (n = 6). Prostate epithelial cells isolated from wild-type (**j,k**) and Tg- *ERG* mouse (**H,I**) prostates develop spheres in semi-solid Matrigel. Spheres grown in the presence of 10 nM DHT on matrigel coated plates developed duct like structures (**H**). Duct like structures was not observed in spheres developed from Tg-ERG in the presence of DHT (**I**). Both wild-type and Tg-*ERG* prostate spheres grown on matrigel in the absence of DHT showed only about 31.8–34% cell survival upon 6 Gy radiation (**J**). Spheres developed from Tg-*ERG* in the presence of DHT showed 52.5% cell survival compared to 20.9% of wild-type derived spheres (**K**). Similarly, LNCaP-LnTE3 cells grown in the presence of 0 to 0.5 ug/ml of doxycycline to induce ERG displayed relatively 2.75 to 3.75 folds enhanced survival compared to controls with 3 and 6Gy radiation (**L**).

To address the question if Tg-*ERG* mice prostates are enriched in prostate progenitor cells, we performed immunofluorescence for cytokeratins CK5 (basal) and CK8 (luminal) markers (Fig. 8A,B). The luminal cells in the prostate glands of 12 months-old Tg-*ERG* mouse showed expression of both cytokeratins CK5 and CK8 (Fig. 8B) suggesting that these double positive cells were basal-intermediate cells. Increased presence of CK5/CK8-positive basal intermediate cells was also observed in prostate specific *AR* conditional knock-out mouse^36^. To assess potential effects of ERG on differentiation block in surviving luminal epithelial cells of Tg-*ERG* prostates, we isolated total cells from ventral prostate glands of 12 months-old Tg-*ERG* mice and wild-type litter mate controls and analyzed for cell lineage (Lin-) markers (CD31, Ter119, CD45) or other cell type markers (Sca1, CD49f and CD133) by flow cytometry (Fig. 8C,D). Our analysis showed that Tg-*ERG* mouse ventral prostates had about 4 fold increase of viable Lin- cell population (Fig. 8E). Lin^neg^ CD49f ^neg/l^°^w^ Sca1^med^ population of cells exhibited overexpression of basal/mesenchymal marker, Sca1 and down regulation of CD133 (Fig. 8F). Increased presence of this cell population in Tg-*ERG* mouse prostates suggested their identity as intermediate cells, which were partially differentiated and shared characteristics of both basal and luminal cells. Hence, we performed an *in vitro* prostate sphere assay^37, 38^ to determine if these cells have ability to form prostate spheres/colonies. Epithelial cells from the prostate gland of 3- to 13-month-old Tg-*ERG* mice showed increased sphere forming capability than wild-type littermates (Fig. 8G). Spheres formed from wild-type mouse appeared more differentiated upon the addition of the androgen hormone, DHT (Fig. 8H). However, the spheres derived from *ERG*-transgenic mouse showed foci without development of duct like structures, a hallmark of compromised terminal differentiation (Fig. 8I).

### Induced expression of ERG confers and resistance to cell death and selective cell proliferation

To examine the cells with ER stress phenotype induced by ERG have survival capability, we isolated prostate cells from ventral prostate glands, grown as prostate spheres in the presence or absence of hormone, DHT for 48 hrs and then subjected to 0, 3 and 6 Gy radiation and spheres were maintained in culture for 7 days (Fig. 8J,K). In the post-radiation short-term cultures, Tg-*ERG* spheres displayed enhanced cell survival in the presence of DHT compared to wild-type prostate spheres (Fig. 8K), suggesting that expression of ERG may provide survival/growth advantage to surviving luminal cells. To further confirm these findings in human prostate cancer culture system, LNCaP-Ln*TE3* with or without ERG induction were subjected to 0, 3 and 6Gy radiation followed by continued culture for 14 days. Consistent with sphere cultures from the Tg-*ERG* mice, ERG positive LNCaP-Ln*TE3* cells showed enhanced cell survival (Fig. 8L). Overall the observations of increased survival of sphere cultures indicate the critical role of ERG in enriching prostate luminal epithelial cells of the Tg-*ERG* mice with adaptive survival.

**Figure 9:**
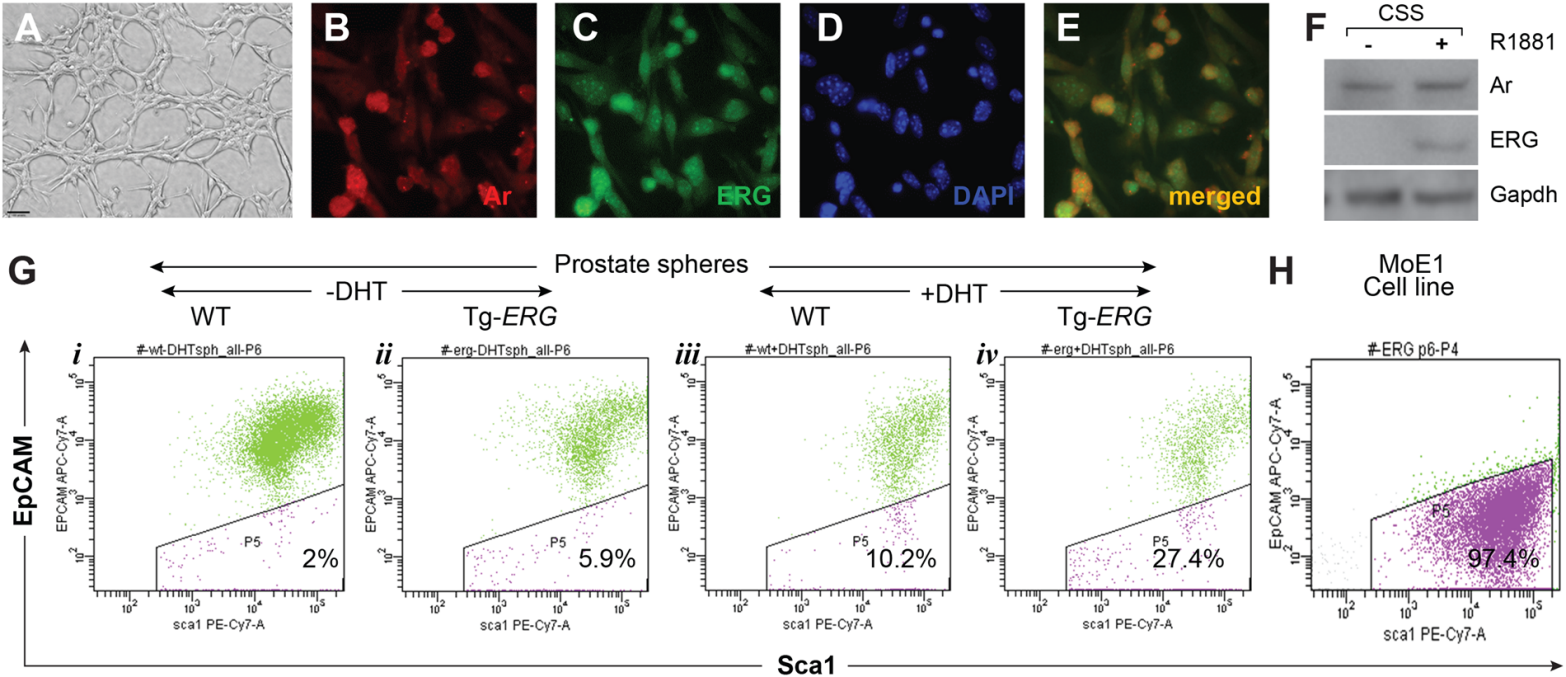
Spontaniously immortalized Tg-*ERG* mouse prostate epithelial cell line and epithelial cells isolated from Tg-*ERG* derived prostate spheres show similar features. Spontaniously immortalized Tg-*ERG* mouse prostate epithelial cell line, MoE1 (p6) grown in PrEGM supplemented with 1nM R1881 (**A–E**) show expression of both Ar (**B**) and ERG (**C**) by immunofluorescence using AR and ERG antibodies. Dapi (**D**) and Ar + ERG merged images (**E**) show colocalization of Ar and ERG in the muclei of MoE1 cells. Western blot analysis of MoE1 cells grown in charcoal stripped serum containing media show the expression of ERG upon induction with 1 nM R1881 (**F**). FACS analysis based on EpCAM of cells dissociated from the prostate spheres grown from wild-type, Tg-*ERG* mouse prostates in the absence (**G** *i, ii*) and presence of 10 nM DHT (**G** *iii, iv*) showabout 2–3 fold increase in EpCAM negative cell population (10.2% wt vs 27.4% Tg-*ERG* spheres). Consistant with prostate spheres, MoE1 cells (**H**) show increasing number of EpCAM (~97%).

Since the cell surface marker analysis revealed the presence of unique “skewed population of luminal cells with increased sphere forming capability, we attempted to establish cell lines from Tg-*ERG* mouse prostates. We could atleast establish 4 independent clones that were spontaneously immortalized with very low proliferation rate. Our initial characterization of a cell line MoE1 (mouse derived epithelial cells) grown in PrEGM (Fig. 9A) supplemented with 1 nM R1881 showed the expression of ERG and Ar by immunofluorescence (Fig. 9B–E). These cells also have shown the expression of both Ar and ERG proteins by Western blot analysis (Fig. 9F). To further characterize the the population of cells that may contribute to the increased proliferation (sphere forming capacity) in the mouse prostate glands, we performed 6 color flow cytometry for Lineage markers (CD31, TER119, CD45), stem cell markes CD49f, EpCAM, Sca1, CD133 and DAPI of ERG prostate derived spheres Tg-*ERG* and wild-type mice. Majority of cells from Tg-*ERG* derived spheres were positive for stem cell markers CD49f and Sca1. However staining with EpCAM revealed about 3 fold increase in the EpCAM negative population of ERG prostate spheres (Fig. 9Gi–iv). Analysis of MoE1 cell line showed that majority of cells (97.1%) were positive for CD49f and Sca1, but negative for EpCAM (Fig. 9H).

## Discussion

Since, over half of the prostate cancer patients harbor *TMPRSS2-ERG* fusions, understanding the mechanistic role of ERG in mediating oncogenic transformation of prostate epithelium would facilitate better therapeutic strategies. Differentiated prostate epithelial cells secrete prostatic fluids, one of the components of semen through ER mediated transport mechanism. Under normal conditions, ER orchestrates the synthesis; folding and transport of at least one third of the proteins that mediate crucial signaling roles such as cell surface receptors, transporters, or polypeptide hormones. Factors such as nutrient deprivation, hypoxia, and point-mutations in secreted proteins lead to the accumulation of misfolded proteins or aggregation within the ER, namely ER stresses. Accumulation of unfolded or misfolded proteins by these factors in the ER lumen alters the ER homeostasis and activates UPR. As cancer usually arises and progresses in a stressed cellular microenvironment, transformed cells use UPR activation as a pro-survival strategy^39^.

**Figure 10:**
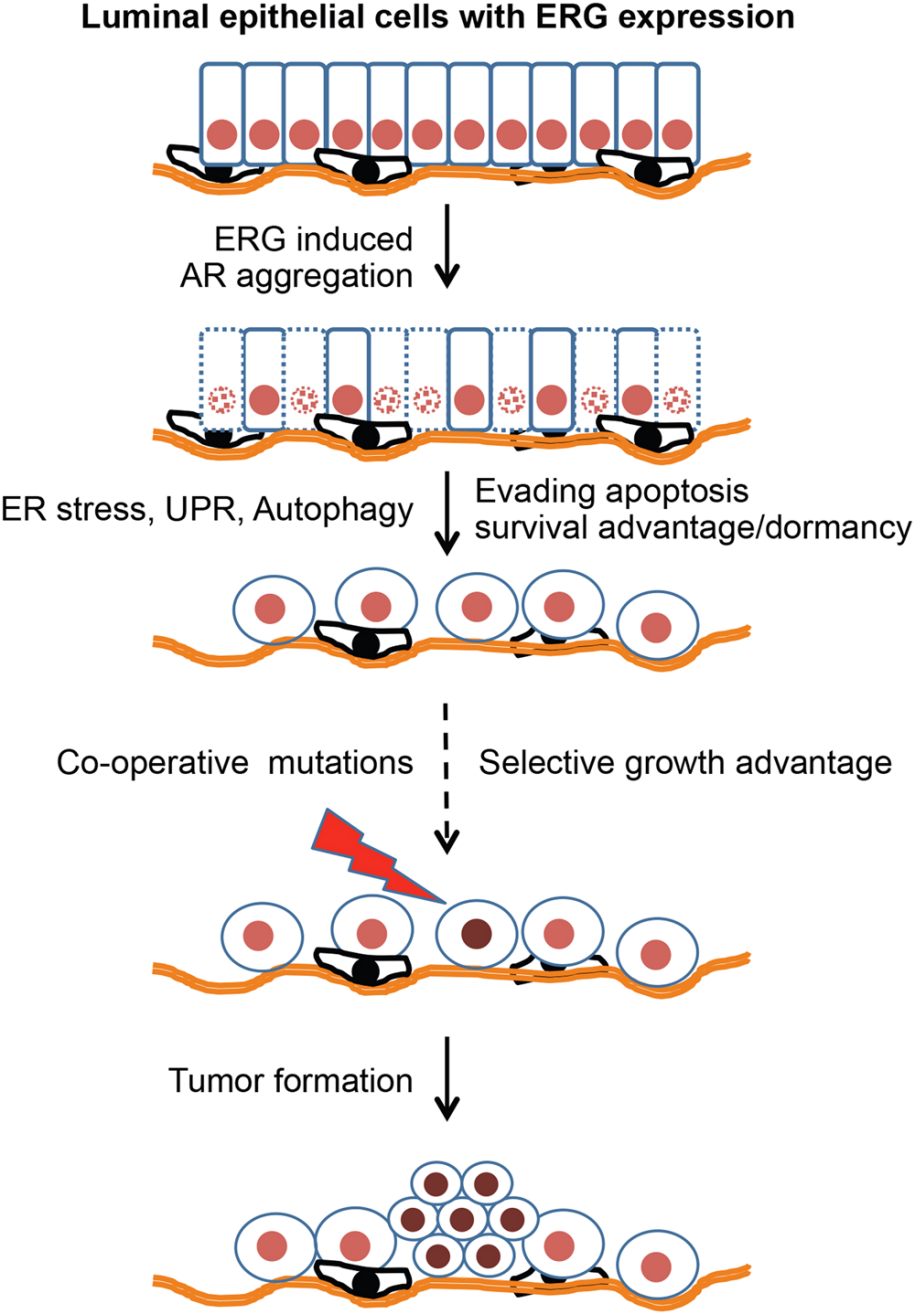
Model depicting ERG-induced AR aggregation as one of the key molecular pathways towards malignant transformation of prostate luminal epithelial cells. Significant numbers of differentiated luminal epithelial cells initially undergo apoptosis due to transgenic expression of ERG and later gain a survival advantage. During this transition, several proteins involved in ER stress and autophagy pathways were up-regulated in luminal epithelial cells. These cells potentially serve as targets for subsequent genetic alterations that confer proliferative potential.

Our data show that ERG induces ER stress and, as a consequence, the canonical pathways of UPR were found differentially regulated towards cell survival. Histological analysis of Tg-*ERG* prostate glands revealed a transient upregulation of apoptotic cell death around 6 months. However, the luminal surface of the older Tg-*ERG* mouse prostate glands contained sparsely organized cells with subtle abnormal morphology with PIN lesions. We observed a slight increase in the cell proliferation as evidenced by increased ki67 staining in Tg-*ERG* mice over 30 months of age. Of note, we observed the presence of ballooned ER in Tg-*ERG* mouse prostate cells from about 5 months of age, with several abnormal sub-cellular organelles suggests ER stress (Fig. 2B,D). This observation was substantiated by the expression analysis of ER stress transcription factors (Fig. 3A,B) and 3 key sensors of ER stress pathway (Fig. 4A). Since the protein overload of ER due to potentially impaired protein folding or misfolding are well established in the induction of stress, we reasoned to examine the upstream events that induce ER stress. Several studies have shown that AR protein is aggregated due to either poly glutamine expansion or increased hormone^30, 31^. Our novel observations have shown that in the presence of androgen, ERG induced AR aggregation. Crucial findings of this study include the identification of ERG induced AR aggregation may have attenuated AR function. Decreased levels of Ar protein in Tg-*ERG* mouse prostates may refelect increased aggregation and decreased monomers as seen in aggregation assay. The presence of *in situ* physical interaction between ERG and AR may be a critical step to drive AR protein misfolding/aggregation. Although the precise contact points on AR for ERG interaction have not been well characterized, the ETS domain of ERG was shown to interact with AR in DNA independent manner^28^. It is possible that the reprogramming of AR cistrome by ERG^19^ may reflect AR tolerance/adaptaion for ERG subsequent to initial events of ERG/AR driven cellular stress as noted in this report. Our results suggest that the terminally differentiated luminal epithelial cells with ERG expression undergo apoptosis due to ERG-induced ER stress likely resulting form AR misfolding/aggregation. However, either upon continued expression of ERG, a subpopulation of cells of the luminal surface with altered expression of cell surface markers evades cell death due to activation of cell survival pathways (Fig. 10). Overall, our findings along with recently demonstrated activation of YAP1 of Hippo pathway by ERG in the development of age-dependent adenocarcinoma^20^ may be one of the mechanisms and suggests for a critical role for ERG in early pro-tumorigenic events leading to progression/development of prostate cancer in humans. Taken together, new insights from this report enhance the biochemical and biological role of ERG in early stages of prostate cancer development and its utility both as biomarker and therapeutic target.

## Materials and Methods

### Analysis of Tg-*ERG* transgenic mice

ERG transgenic mice Tg(*Pbsn-ERG*)*1Vv* (referred in this manuscript as Tg*-ERG*) were obtained from Dr. Valera Vasioukhin, Fred Hutchinson Cancer Research Center. The mice are maintained according to Uniformed Services University of the Health Sciences- Institutional Animal Care and User Committee (USU-IACUC) approved animal study protocols for breeding (SUR-12-597 and SUR-15-597) and research (SUR-12-581 and SUR-15-581). All experimental methods performed in this manuscript were conducted in accordance with the relevant guidelines and regulations. Mice were euthanized using CO_2_ asphyxiation, and prostate tissues were procured for either paraffin embedding or frozen storage for histological and molecular analyses using established protocols.

### Histology

Wild-type and Tg*-ERG* transgenic male mouse ventral prostates were collected at different time points and processed for paraffin embedding and sectioning. The sections were stained with H&E for general histology and were scanned using a Nano Zoomer Digital Pathology system, Hamamatzu Corporation, at 40x magnification and analyzed using NDP View software.

### Antibodies and plasmids

ERG (9FY, Biocare Medical Inc., CA, USA); ERG (EPR 3864; Cat No. 2805-1, Epitomics, USA); AR (#sc815, Santa Cruz Biotechnology, Inc., Dallas, TX, USA); ER Protein Folding Antibody Sampler Kit (#4759), ATF6, PERK, IRE1α, GAPDH (Cell Signaling Technology, Danvers, MA, USA); Nkx3.1 was a kind gift from Dr. Bieberich, UMBC, Baltimore, MD, USA and Tmprss2 was a kind gift from Dr. Lin, UMAB, Baltimore, MD, USA. pEGFP-C1-AR was a gift from Michael Mancini (Addgene plasmid #28235).

### Immunoblot analysis

Total protein lysates were prepared in the MPER buffer (Biorad) supplemented with protease inhibitor cocktail and phosphatase inhibitors (Thermo Scientific, Waltham, MA) and westerns were performed as per standard protocols. The antibodies used were ERG (9FY 1:500; AR (1:2000; ATF6, PERK, IRE1α, Bip/GRP78, GRP92, ERp57, alpha-tubulin (1:1,000) and GAPDH (1:5,000).

### Immunohistochemistry

Slides were deparaffinization in xylene, sequentially rehydrated in a descending series of ethanol concentrations followed by antigen retrieval in citrate buffer (pH 6.0) for 30 minutes. Endogenous peroxidase was blocked with 3% H_2_O_2_ for 15 min at room temperature. Primary antibodies diluted in 1xPBS were incubated overnight at 4 °C [ERG mouse monoclonal antibody (9FY-CPDR, 1:100), androgen receptor C-20 (sc815, 1:2000), Nkx3.1 (1:1000), and Tmprss2 (1:1000), GRP78 (1:1000), PDI (1:1000)]. Following the addition of appropriate secondary antibodies, the immune reactivity was visualized using DAB as chromogene (Vector #SK4100) and counterstaining with hematoxylin. Slides were then dehydrated in graded alcohol washes, cleared using xylene, and mounted for microscopic analysis.

### Immunocytochemistry

Cells were fixed with 4% PFA buffered in PBS, permeabilized with 0.1% Triton X-100 in PBS, and blocked with 1% normal horse serum (Vector Laboratories) before incubating with appropriate primary antibodies. Species-specific secondary antibodies (Alexa Fluor-594 goat anti-mouse and Alexa Fluor-488 goat anti-rabbit (Invitrogen Corp., Carlsbad, CA, USA) were subsequently applied together with DAPI. F-actin was stained with Alexa Fluor-594 phalloidin (Invitrogen Corp., Carlsbad, CA, USA).

### Electron microscopy (EM)

Prostate tissues were fixed in Kornovsky buffer (#15720, Electron Microscopy Sciences, Hatfield, PA, USA) overnight at 4 °C and washed in the same buffer for 30 min followed by post-fixation for 2 h at 4 °C in 2% Osmium tetroxide in cacodylate buffer. After fixation, the material was dehydrated through a graded series of alcohols and embedded in SpurrResin (Electron Microscopy Sciences, Hatfield, PA, USA). Samples were then polymerized at 75 °C for 12 h and sections were cut on a Leica Ultracut UC6 ultramicrotome (Leica Microsystems, Buffalo Grove, IL, USA). Copper grids containing thin sections were post-stained for 20 min in 2% uranyl acetate and 5 min in Reynold’s lead citrate before observation in a JEOL JEM-101transmission electron microscope (JEOL USA, Inc., Peabody, MA, USA). Images were captured on an AMTXR50S-A digital camera. For light microscopic analysis, semi-thin sections (2 μm) were stained with Toluidine blue.

### ER stress Transcription Factor Activity Profiling Assay

Analysis of 16 ER stress/UPR related TFs was performed according to the manufacturer’s instructions using the ER Stress (UPR) TF Activation Profiling Plate Array FA-1006 (Signosis). Nuclear proteins were prepared from 3.5 and 10 months-old Tg-ERG mouse prostates and their age matching littermate controls, LNCaP-LnTE3 cells without or with doxycycline induction were prepared according to the manufacturer’s recommendations using Signosis Nuclear Extraction Kit from Signosis (SK-0001).

### *In situ* Proximity Ligation Assay

ERG and AR interactions were detected using Duolink^®^ *In Situ* Red Starter Kit Mouse/Rabbit (#DUO9201, Sigma-Aldrich, St. Louis, MO, USA). Briefly, LNCaP-LnTE3 cells grown on glass cover glass with and without ERG induction by doxycycline were washed twice in PBS, fixed for 10 min in 4% (w/v) paraformaldehyde in PBS, permeabilized for 10 min with 0.3% (v/v) Triton X-100 in PBS and blocked for 1 h with Sigma PLA blocking buffer at 37 °C. The cells were incubated with the indicated antibody pairs (mouse anti-ERG [CPDR, #9FY at 1:8000 dil] and rabbit anti-AR [Origene Technologies, Inc., Rockville, MD USA]) # TA3076387, 1:2000 dil]) at 37 °C. Secondary antibodies conjugated to PLUS and MINUS probes, ligation and amplification steps were performed according to manufacturers instructions. Images were acquired by Zeiss AxioImager. M2 microscope and StereoInvestigator 9 software.

### AR aggregation assay

AR aggregate filter-trap assay was performed according to Wanker *et al*.^32^ with modifications. LNCaP cells with and without ERG induction were lysed in 200 ul cold lysis buffer (50 mM Tris-HCl, pH 7.4, 100 mM NaCl, 0.5% Nonidet P-40, 1 mM EDTA, and protease inhibitor mixture (Active Motif, Carlsbad, CA, USA) and centrifuged for 5 min at 14,000 rpm. Each pellet was resuspended in 100 ul DNase buffer and digested with 0.5 mg/ml of DNase I at 37 °C for 1 hr. The aliquots corresponding to 50–100 *μ*g of protein were filtered through 0.2 *μ*m Cellulose Acetate and 0.45 *μ*m Nitrocellulose membranes. Membranes were blocked in 5% NFD and captured aggregates were detected by immunoblotting with AR antibody and visualized using the ECL Plus kit (GE Healthcare Life Sciences, Pittsburg, PA, USA).

### Prostate sphere formation assay

Age matched wild-type and Tg-*ERG* transgenic mouse prostates were minced into small pieces in DMEM media supplemented with 10% FBS and incubated in a final concentration of 1mg/ml of type I collagenase solution (Invitrogen, USA) for 60–90 min as described previously^38^. Prostates Cells were then pelleted, resuspended, and incubated in 0.05% trypsin/EDTA for 5 min at room temperature and passed through 19- to 27-gauge needles. Cells were passed through a 40-ml cell strainer and centrifuged for 6 min at 1,000 rpm. Cell pellet was resuspended in 1 ml of PrEGM and about 10,000 cells were suspended in 1:1 Matrigel (BD Pharmingen, Franklin Lakes, NJ, USA)/PrEGM in a total volume of 100 μl in triplicate and plated around the rims of wells in a 12-well plate. After the Matrigel solidified, 1 ml of PrEGM was added and the plates were stored in a CO_2_ incubator at 37 °C. Medium was replenished every 3 days. Ten days after plating, spheres with a diameter over 40 μm were counted.

### Co-immuno-precipitations of ERG and AR

HEK-293 and HEK 293-LTE3 (HEK 293 with ERG) cells grown in serum starvation conditions (in 10% charcoal stripped serum) were transfected with 12 µg of pCMV-3MYC-AR (full length androgen receptor), pCMV-3MYC-AR:1-565, pCMV-3MYC-AR:566-919, pCMV-3MYC-AR:1-670, and pCMV-3MYC-AR:1-608 plasmids (Hoang, 2014, Mol Cell Ther). Cells were then treated with 1 nM of R1881 24 h after transfection incubated for another 24 h. Cell were lysed in Lysis buffer (50 mM Tris pH 7.9, 5 mM EDTA, 150 mM NaCl, 1% NP-40, and 1 mM Sodium orthovanadate) containing Halt protease and phosphatase inhibitors (Thermo). ERG and myc-tagged AR proteins in the cell lysates were immunoprecipitated with 4 µg anti-ERG rabbit monoclonal (Abcam EPR3864) pre-bound to Protein A/G Plus Ultralink agarose beads (Thermo Scientific) and with 30 µl of Anti-c MYC Agarose (Thermo Scientific), respectively by incubation for 4 hrs. The beads were washed three times for 15 min each in Wash buffer (10 mM Tris pH 7.9, EDTA 0.5 mM, 150 mM NaCl, 0.1% NP-40 with 1x Halt protease and phosphatase inhibitors). The cell lysates were separated on 4% to 12% Bis-Tris gels (Thermo Scientific) and transferred electrophoretically to polyvinylidene fluoride membrane (Thermo Scientific). The membranes were blocked by using ReliaBLOT® Block reagent (Bethyl Laboratories) in Tris-buffered saline and Tween 20 (TBST, 150 mM NaCl, 0.1% Tween 20, 50 mM Tris pH 8.0). Primary antibodies used for immunoblotting were anti-ERG rabbit MAb (Abcam) and rabbit polyclonal MYC-tag antibody (Cat# 06-549, EMD Milipore). The immuno-reaction was detected by using anti-rabbit ReliaBLOT® HRP conjugate (Bethyl Laboratories) for 60 minutes prior to developing with ECL substrate solution (GE Healthcare) and exposure to film.

### Footnotes

The views expressed in this manuscript are those of the authors and do not reflect the official policy of the Department of the Army, Department of Defense or the US Government.

## Electronic supplementary material

Supplementary figures S1-S7
