# Erratum: *ETS* Related Gene mediated Androgen Receptor Aggregation and Endoplasmic Reticulum Stress in Prostate Cancer Development

**DOI:** 10.1038/s41598-017-09612-4

**Published:** 2017-09-06

**Authors:** Taduru L. Sreenath, Shiela S. Macalindong, Natallia Mikhalkevich, Shashwat Sharad, Ahmed Mohamed, Denise Young, Talaibek Borbiev, Charles Xavier, Rishita Gupta, Muhammad Jamal, Kevin Babcock, Shyh-Han Tan, Marja T. Nevalainen, Albert Dobi, Gyorgy Petrovics, Isabell A. Sesterhenn, Inger L. Rosner, Charles J. Bieberich, Peter Nelson, Valeri Vasioukhin, Shiv Srivastava

**Affiliations:** 1 Center for Prostate Disease Research, USU Walter Reed Department of Surgery, Uniformed Services University of the Health Sciences, Bethesda, MD USA; 2 MCW Cancer Center, Medical College of Wisconsin, Milwaukee, WI USA; 3 Department of Genitourinary Pathology, Joint Pathology Center, Silver Spring, MD USA; 4 Department of Biological Sciences, University of Maryland Baltimore County, Baltimore, MD USA; 5 Division of Human Biology, Fred Hutchinson Cancer Research Center, Seattle, WA USA; 6 Urology Services, Walter Reed National Military Medical Center, Bethesda, MD USA

*Scientific Reports* **7**:1109; doi:10.1038/s41598-017-01187-4; Article published online 24 April 2017

This Article contains errors in Figure 1, where panels 1B, 1C, 1D, 1E, 1F, and 1G are mislabelled. The correct Figure 1 appears below as Figure 1. The Figure legend is correct.

**Figure 1:**
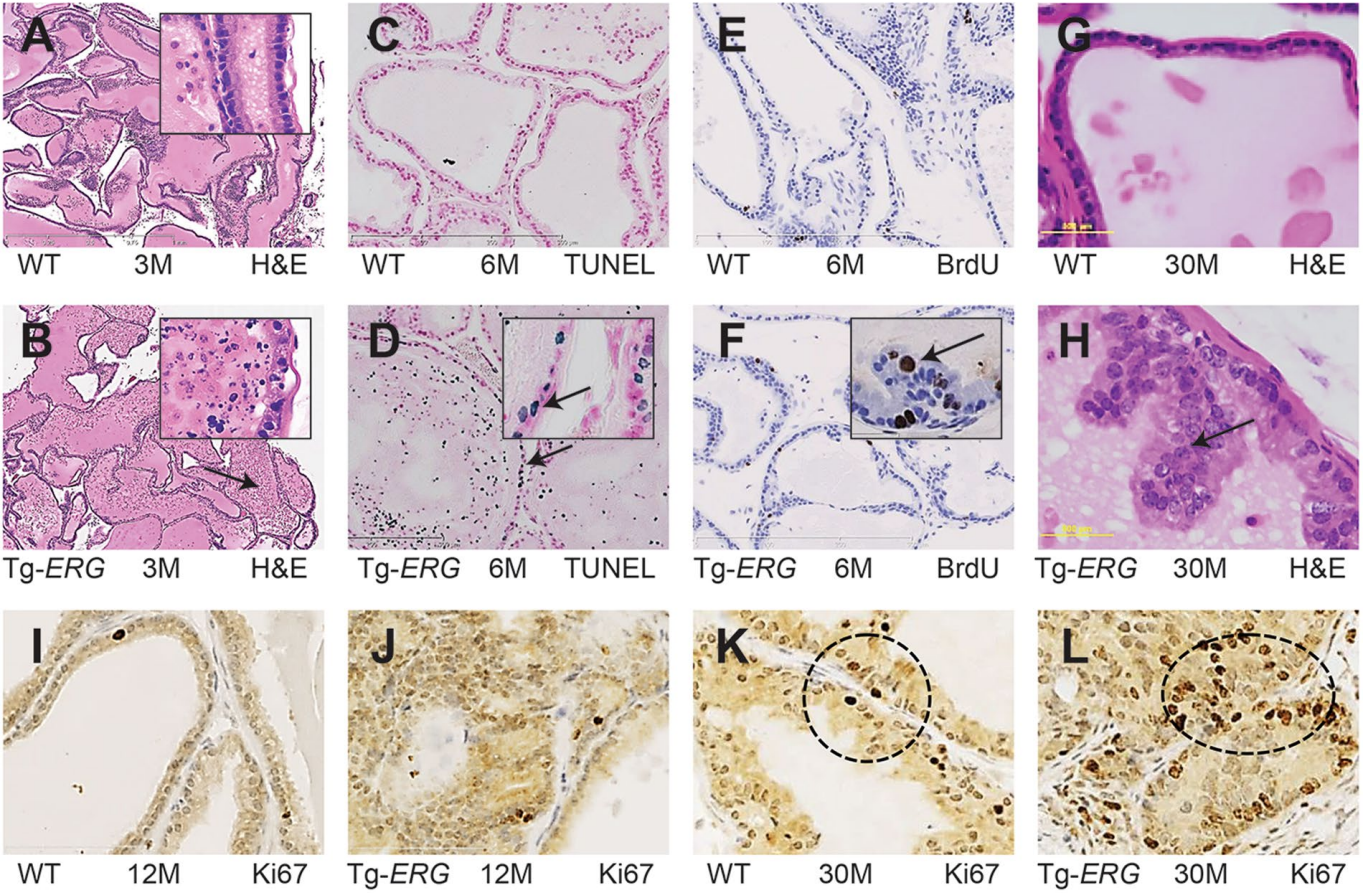
Morphological and histological differences in the ventral prostates of Tg-*ERG* mice. Hematoxylin and eosin staining of prostate glands from 3–30 months-old wild-type (**A,C,G**) and Tg-*ERG* mouse (**B,D,H**) prostate glands. Increased dell death is seen in 3 and 6 months-old display significant cell death (**B,D**). Inserts show nuclear fragmentation and sparsely organized and morphologically distinct luminal epithelial cell layer (note the arrows). TUNEL staining of wild-type (**C**) and Tg-*ERG* (**D**) prostates confirm the cell death due to apoptosis. Short arrow (insert) points to an intact luminal cell undergoing apoptotic cell death while long arrow points to fragmented nuclei of dead cells in the lumen. Cell proliferation analysis has shown an increase in the number of BrdU-positive cells in Tg *ERG* mouse (**F**) than wild-type (**E**). Hematoxylin and eosin staining of 30 month-old transgenic mice display clustered luminal cells resembling high grade PIN lesions (**H**). Similarly, Ki67 immunostaining was also increased in Tg-*ERG* mouse (**J,L**) than wild-type (**I,K**). There is an increase in the Ki67 staining pattern in 30 month-old mouse prostates than 12 month-old prostates (**I–L**). Total number of mice used for each analysis is 5 (n = 5).

