# Supplementary material for: ETS Related Gene mediated Androgen Receptor Aggregation and Endoplasmic Reticulum Stress in Prostate Cancer Development
Source: Sci Rep. 2017 Apr 24;7:1109. doi: 10.1038/s41598-017-01187-4 (PMC5430720; doi:10.1038/s41598-017-01187-4)
Supplement: Supplementary file 1 — Supplementary figures S1-S7 [file 41598_2017_1187_MOESM1_ESM.pdf]

S1

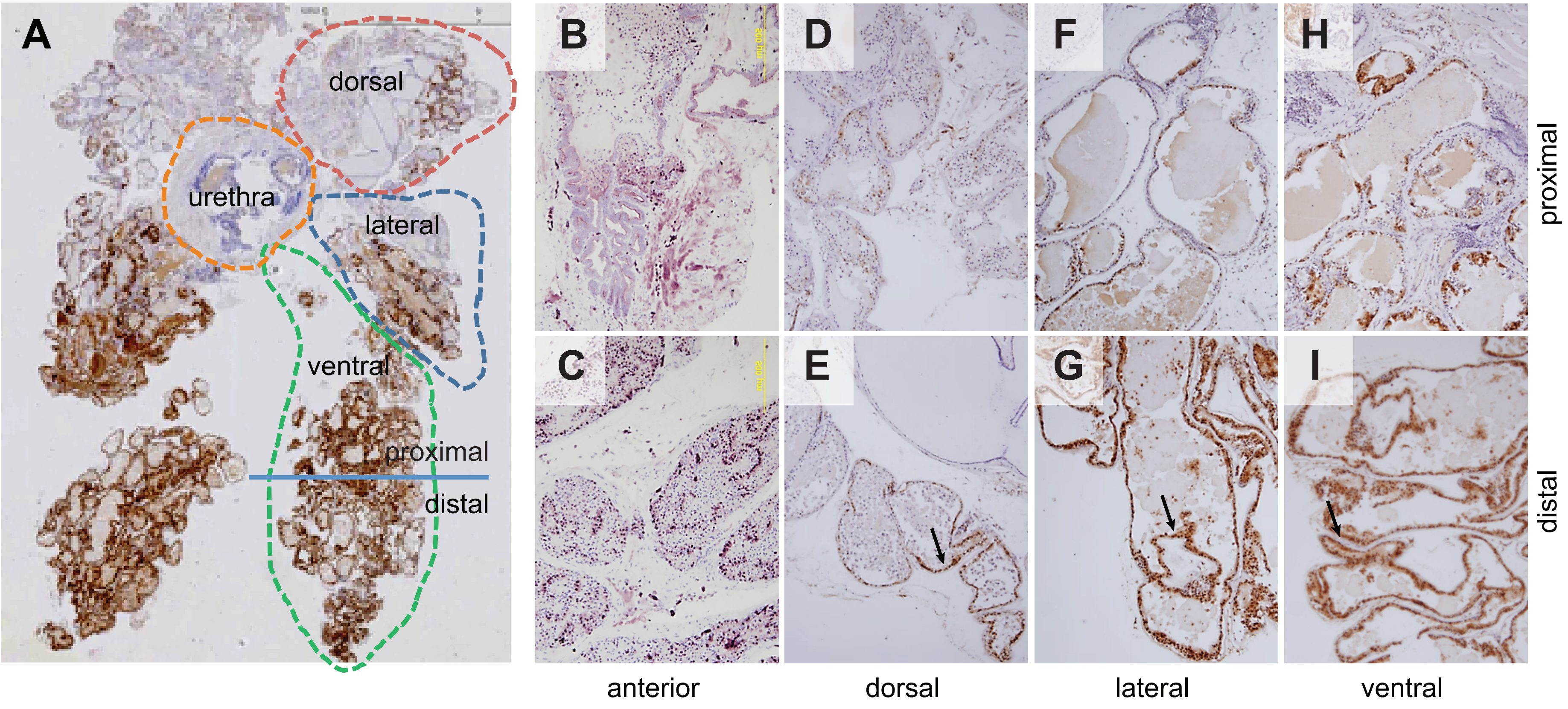

Expression of ERG transgene in 6 month-old Tg-ERG mouse prostate glands. Expression of ERG in the transgenic mouse prostate glands (A) is shown with approximate demarcation of proximal and distal regions. Expression of ERG in proximal and distal regions of anterior (B, C), dorsal (D, E), lateral (F, G) and ventral (H, I) prostates show relative levels. Proximal regions have relatively lower expression than the distal regions. Total number of mice used for each analysis is 5 (n=5).

S2

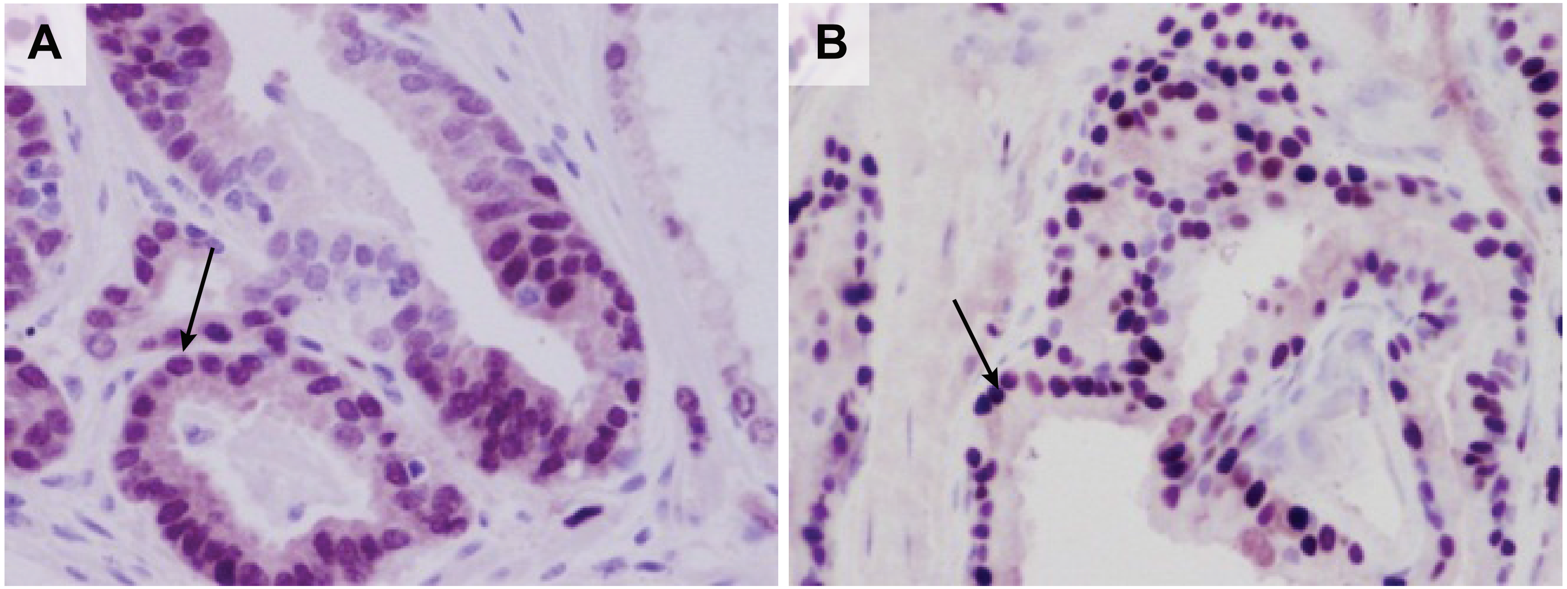

ERG positive human prostate tumor | Tg-*ERG* mouse ventral prostate gland

Immunohistochemical analysis of ERG protein expression in human and in Tg-ERG mouse prostate glands. Relatively comparable levels of ERG were detected in human prostate tumor tissue and (A) the luminal epithelium of transgenic mouse (B) ventral prostate glands. Arrows point to the expression of ERG protein in the nuclei of the luminal epithelial cells by IHC using 9FY antibodies.

S3

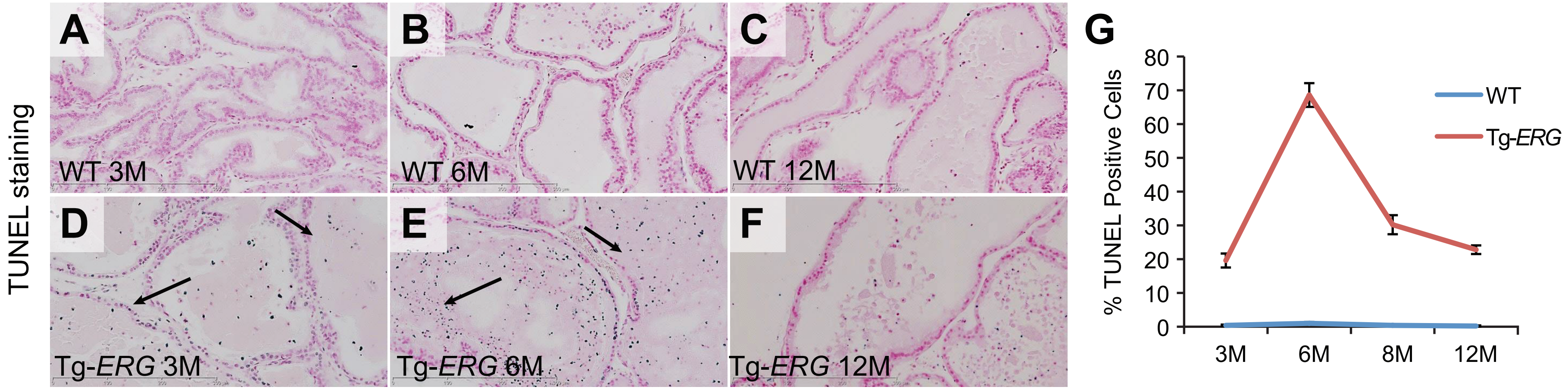

Quantitation of TUNEL staining in ventral prostates of 3, 6 and 12 month-old Tg-ERG mice. TUNEL staining (A-F), an indicator of apoptotic cell death was significantly increased in the Tg-ERG mouse prostate glands (B, 3 months; D, 6 months; F, 12 months) compared to the wild-type (A, 3 months; C, 6 months; E, 12 months). Quantitation of TUNEL staining was represented as percent of TUNEL positive cells in 5 mice and 5sections/mouse (G)

S4

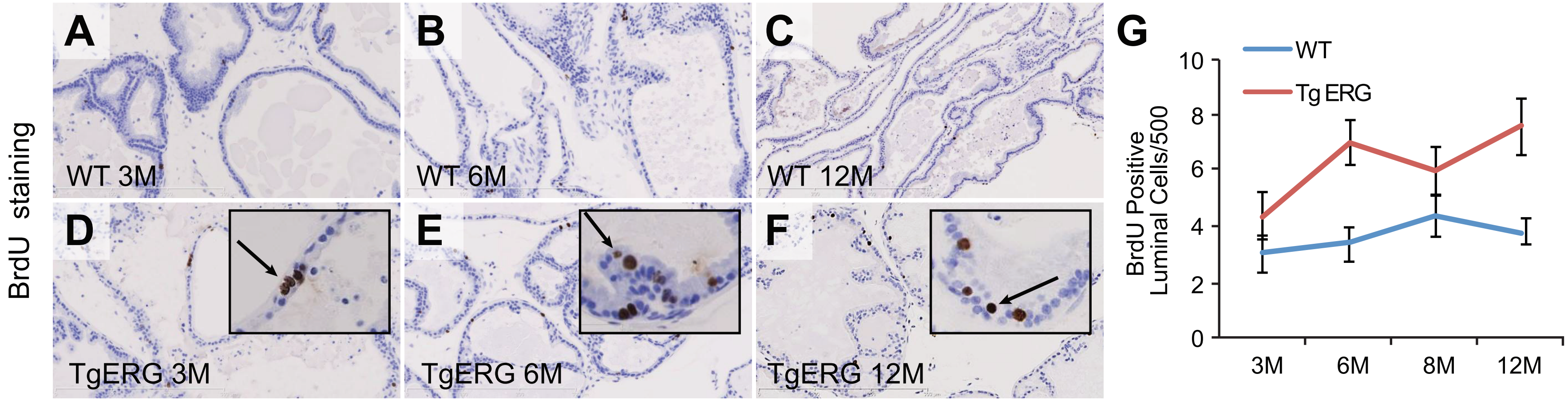

Quantitation of BrdU incorporation in ventral prostates of 3, 6 and 12 month-old Tg-ERG mice. BrdU assay (A-F) an incorporation A F), indicator of cell proliferation has shown relatively slight increase in the number of BrdU-positive cells in Tg-ERG (B, 3 months; D, 6 months; F, 12 months) compared to the wild-type mouse prostate glands (A, 3 months; C, 6 months; E, 12 months). Quantitation of TUNEL staining was represented as percent of BrdU positive cells in 5 mice and 5sections/mouse (G)

S5

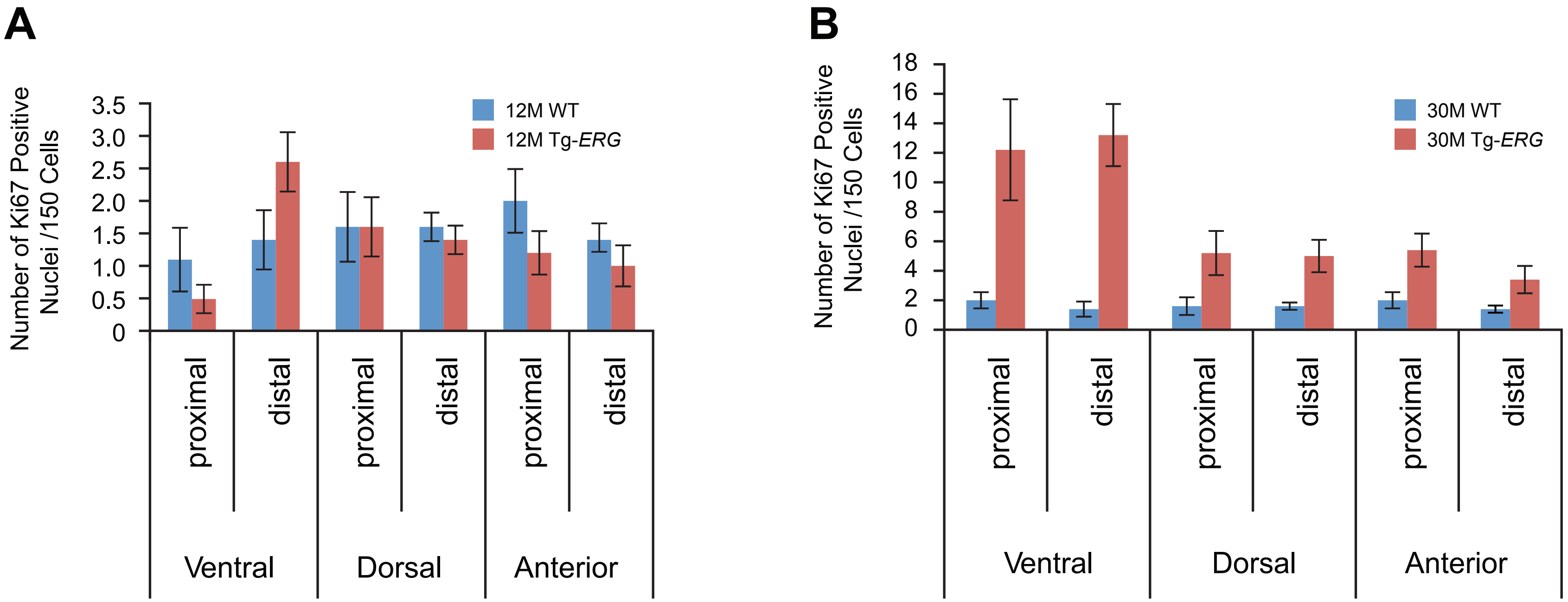

Quantitation of cell proliferation in ventral prostates of 12 and 30 month-old Tg-ERG by Ki67 immunostaining. Immuno-histochemical assay was performed on the proximal and distal regions of anterior, dorsal and ventral prostates of the mice (A, B). Quantitation of Ki67 positive cells /total of 150 luminal epithelial cells from 5 mice and 5sections/mouse was performed and recorded. Significant increase in the proliferation of cells was observed in the distal regions of 12 months-old ventral prostate (A). Both proximal and distal regions of 30 month-old mice showed significant increase in the proliferative marker staining (B).

S6

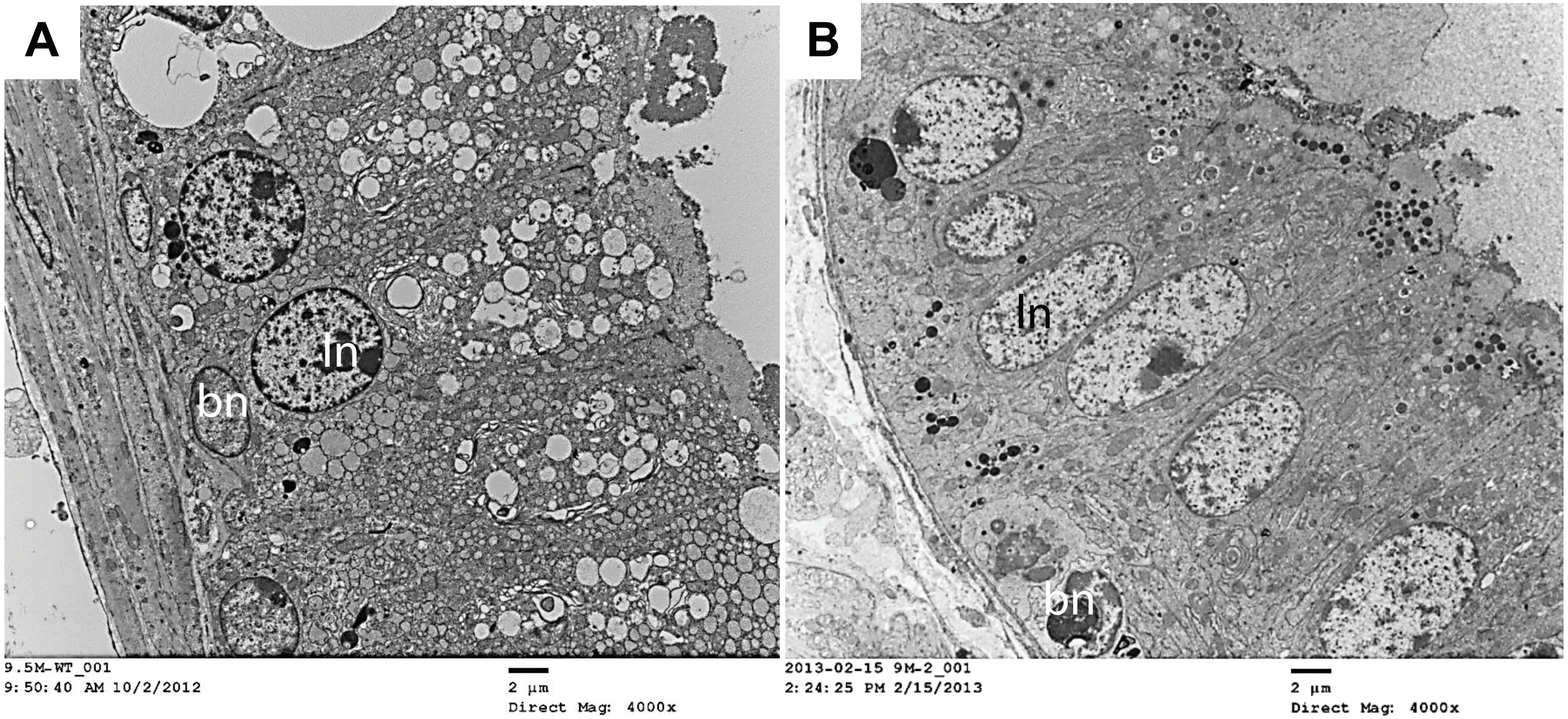

Ultra structural analysis of 9 months-old Lo-MYC transgenic mouse prostate glands. Lo-MYC transgenic mouse ventral prostates do not display the presence of enhanced ER abnormalities (A, WT; B, Lo-cMYC). bn, basal cell nuclei; ln, luminal cell nuclei.

S7

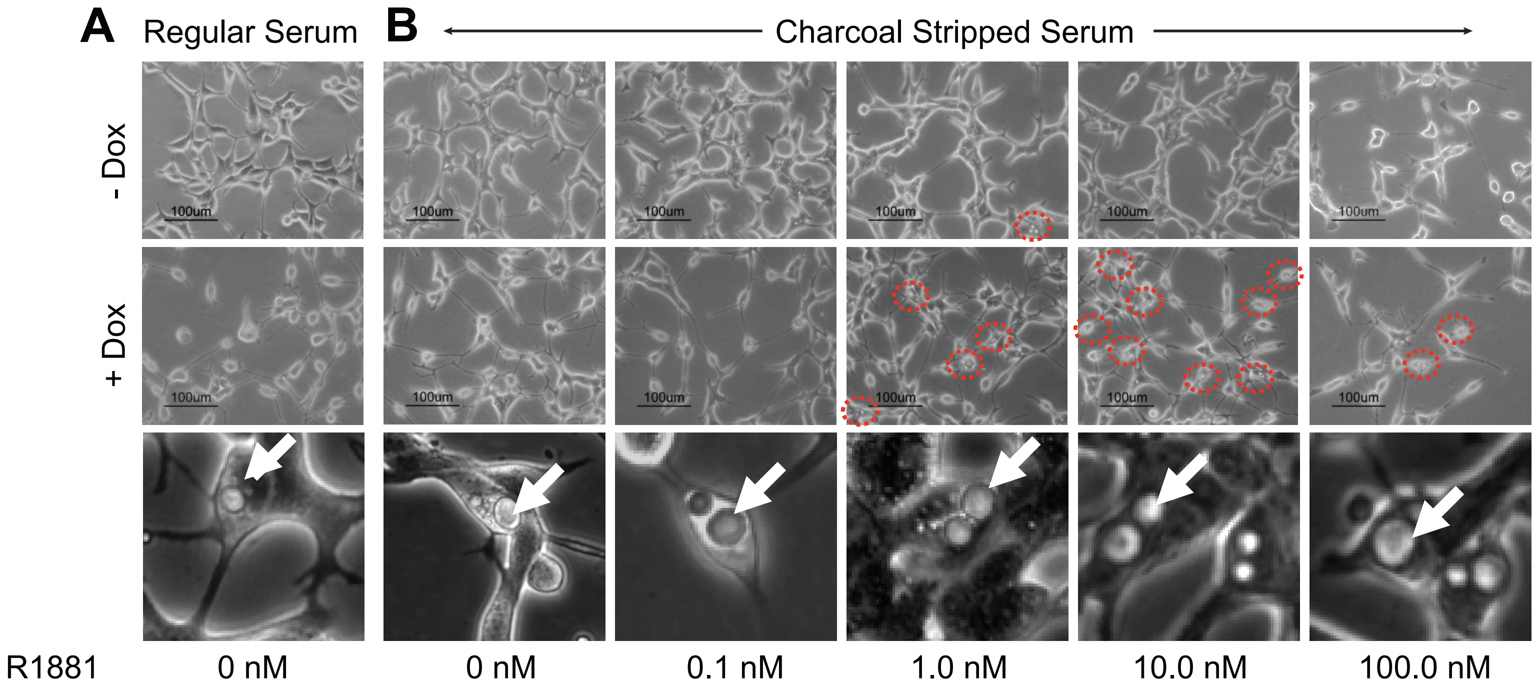

ERG induces ER stress in LNCaP cells. LNCaP-ERG cells were grown in presence of regular (A) and charcoal stripped serum (B) to analyze hormone dependent and independent effects of ERG in the induction of ER stress. Increased numbers of vacuole formations were observed with doxycycline induction of ERG (middle and lower panels) with increasing concentrations of R1881. Higher magnification of the presence of vacuoles was shown in the lower panel with white arrows.
